# Contactless continuous guidance of matter in its main states using curved elongated Bessel-like acoustic fields

**DOI:** 10.1073/pnas.2614959123

**Published:** 2026-10-23

**Authors:** Dmitrii Nikolaev, Denys Iablonskyi, Alexander Martinez-Marchese, Marika Sirkka, Mikel Aldea, Chinedum Okwudire, Edward Hæggström, Asier Marzo, Ari Salmi

**Affiliations:** ^a^ Electronics Research Laboratory, Department of Physics, University of Helsinki, Helsinki 00710, Finland; ^b^ Department of Mechanical Engineering, University of Michigan, Ann Arbor, MI 48109; ^c^ UpnaLab, Department of Statistics, Computer Science and Mathematics, Public University of Navarre, Pamplona 31006, Spain

**Keywords:** contactless manipulation, acoustic levitation, Bessel beams, acoustic vortices, acoustic radiation force

## Abstract

Acoustic manipulation uses sound waves to exert force on matter, enabling contactless movement, trapping, and the structuring of particles in bulk media. It offers a contamination-free approach for handling matter in applications spanning across chemistry, biology, and advanced manufacturing. However, state-of-the-art techniques, including acoustic holograms or phased arrays of transducers (PATs) are limited to transporting individual particles or small clusters one by one, and are not suitable for dispersed matter such as gases or aerosols. Here, we introduce a method for guiding a continuous flow of matter along reconfigurable three-dimensional trajectories using elongated Bessel-like acoustic fields (EBAFs). These fields generate converging radial acoustic forces along a programmable path, forming stable mid-air conduits that continuously transport matter. Compared to existing methods such as near-field acoustic levitation, acoustofluidic separation, particle manipulation with vortex and Airy beams, the proposed method offers enhanced versatility, scalability, and real-time control of transport trajectories in mid-air without requiring physical guiding structures. We developed methods to generate EBAFs with PATs tailored to different particle sizes and material properties, and experimentally demonstrated guidance of solids, liquids, aerosols, and gases along programmable trajectories. This technique enables containerless laboratory operations, precise and contamination-free reagent or energy delivery, and integration into advanced additive manufacturing processes that require controlled distribution of matter in different states to target zones.

## Significance

Acoustic manipulation uses sound waves to move and trap matter without physical contact, providing a contamination-free tool for applications in industry and academia. Existing methods, such as acoustic holograms and phased arrays, are limited to handling individual particles or small clusters and cannot effectively transport dispersed matter, such as gases or aerosols. Here, we introduce a method for the contactless transport of matter in solid, liquid, gas, and plasma states by guiding the flow of matter along reconfigurable three-dimensional paths. The approach employs elongated Bessel-like acoustic fields to form stable mid-air conduits tailored to particle properties. This programmable guidance enables containerless processing, precise delivery of reagents or energy, and opens possibilities for controlled material distribution in advanced manufacturing.

Acoustic levitation utilizes ultrasonic waves to exert forces, enabling the suspension of objects in mid-air without physical contact (1). This technique enables the levitation of a wide range of materials including solids (2, 3), liquids (4, 5), and gases (6), over a wide range of particle sizes, from individual cells (7) to millimeter-sized objects (8), and even centimeter-sized objects exceeding the acoustic wavelength (9).

The broad applicability of acoustic levitation has led to various scientific and industrial uses (10) including material processing in microgravity, biological sample handling (11), drug synthesis (12), fluid sample analysis (13), chemical reagents mixing (14), crystallography experiments (15), and explorations of low-carbon technologies (16).

There are multiple types of acoustic fields for particle levitation, including standing waves (17–19), patterned fields generated using acoustic holographic plates (20, 21) or phased array of transducers (PATs) (22–24), and single acoustic beams (25–28). Among these, PATs are particularly powerful, as they allow dynamic reconfiguration of the acoustic field, enabling translation of levitated particles through space (29).

Dynamic manipulation is typically realized by refocusing or spatially translating the acoustic field over time, thereby moving the trapped particles along a required trajectory. However, this approach transports individual particles or small clusters sequentially, rather than enabling continuous or bulk transport. Achieving continuous transportation of matter, such as a stream of solid particles or fluids along reconfigurable paths, would significantly expand the capabilities and applications of acoustic manipulation.

Several approaches have been developed to enable continuous material transfer. Near-field acoustic levitation allows objects to slide slightly above a vibrating surface (30–34), while surface acoustic waves (SAW) have also been used for the manipulation and transport of micro- and nanoscale objects (35). In acoustofluidic systems, particle separation and transport are realized through the combined action of acoustic radiation forces and streaming vortices generated by interdigitated transducers, which guide particles along predefined tracks (33, 36, 37). Bulk manipulation can also be achieved using structured beams, such as vortex beams (38–40) and Airy beams (41, 42), which enable long-range particle guidance in fluid media.

However, these techniques generally rely on solid, vibrating surfaces that define fixed, nonreconfigurable particle trajectories, which limits their flexibility for dynamically programmable applications. Acoustic vortices have been shown to focus powders in free space without physical boundaries (38), and can also be used for programmable rotation and translation (43) but the resulting fields are static. Similarly, Airy and vortex beams have been used to create curved trajectories for microbubbles in water using a combination of reflective surfaces and holographic lenses (39–42). These approaches, however, are constrained by the need for buoyancy and viscous damping, which restricts their applicability to specific media (e.g., water) and particle types (e.g., bubbles, plastics). Furthermore, the generated acoustic fields are not dynamically reconfigurable, and the particle trajectories remain fixed.

PATs can significantly enhance the versatility and enable the dynamic reconfigurability of continuous transport of matter. Numerical studies have demonstrated the feasibility of generating curved elongated acoustic vortices using bowl-shaped PATs (44) as well as curved elongated focused Bessel beams with flat PATs (45–47). The latter approach has been shown to provide laminar air-channel guidance. However, mist guidance in this case relies on laminar streaming from the mist source and remains highly susceptible to turbulence.

In this work, we present a method for the continuous guidance of matter in its main states (solid, liquid, gas, and ionized gas) along reconfigurable three-dimensional trajectories. This was achieved by using channel-shaped traps based on a family of elongated Bessel acoustic fields (EBAFs). EBAFs generate converging radial forces along their path, effectively confining matter within a prescribed trajectory. To generate such fields, we developed a method for calculating the drive signals for the PAT. For each type of matter and particle size, a suitable field configuration was determined to ensure stable and efficient guidance. Furthermore, real-time field reconfiguration of the PAT emission allowed for on-demand adjustment of the guidance pathways. Using this approach, we experimentally demonstrated continuous and programmable guidance of solid particles, droplets, and streams of liquids, aerosols, gases, and even ionized hot gas, along arbitrary 3D paths using different EBAF modes.

The proposed method for continuous matter guidance offers a versatile and contamination-free tool for applications ranging from fundamental science to industrial manufacturing. The method can be integrated into modern additive manufacturing processes, including droplet-based bioprinting (48), drop-on-demand using molten metal (49), and powder delivery in directed energy deposition (DED) (50). As demonstrated in this work, EBAFs can steer gas and aerosol flows, which can be particularly beneficial in atmospheric pressure chemical vapor deposition (APCVD) or in processes employing electrospray ionization (ESI), such as liquid chromatography–mass spectrometry (LC–MS) systems. Furthermore, since ultrasonic standing waves have been shown to block, focus, or separate ion beams (51), EBAFs could potentially be adapted for precise ion beam guidance. These examples illustrate only a fraction of the broad spectrum of potential applications made possible by the proposed approach.

## Results

### Generation of EBAFs for Guiding Matter.

**Fig. 1.**
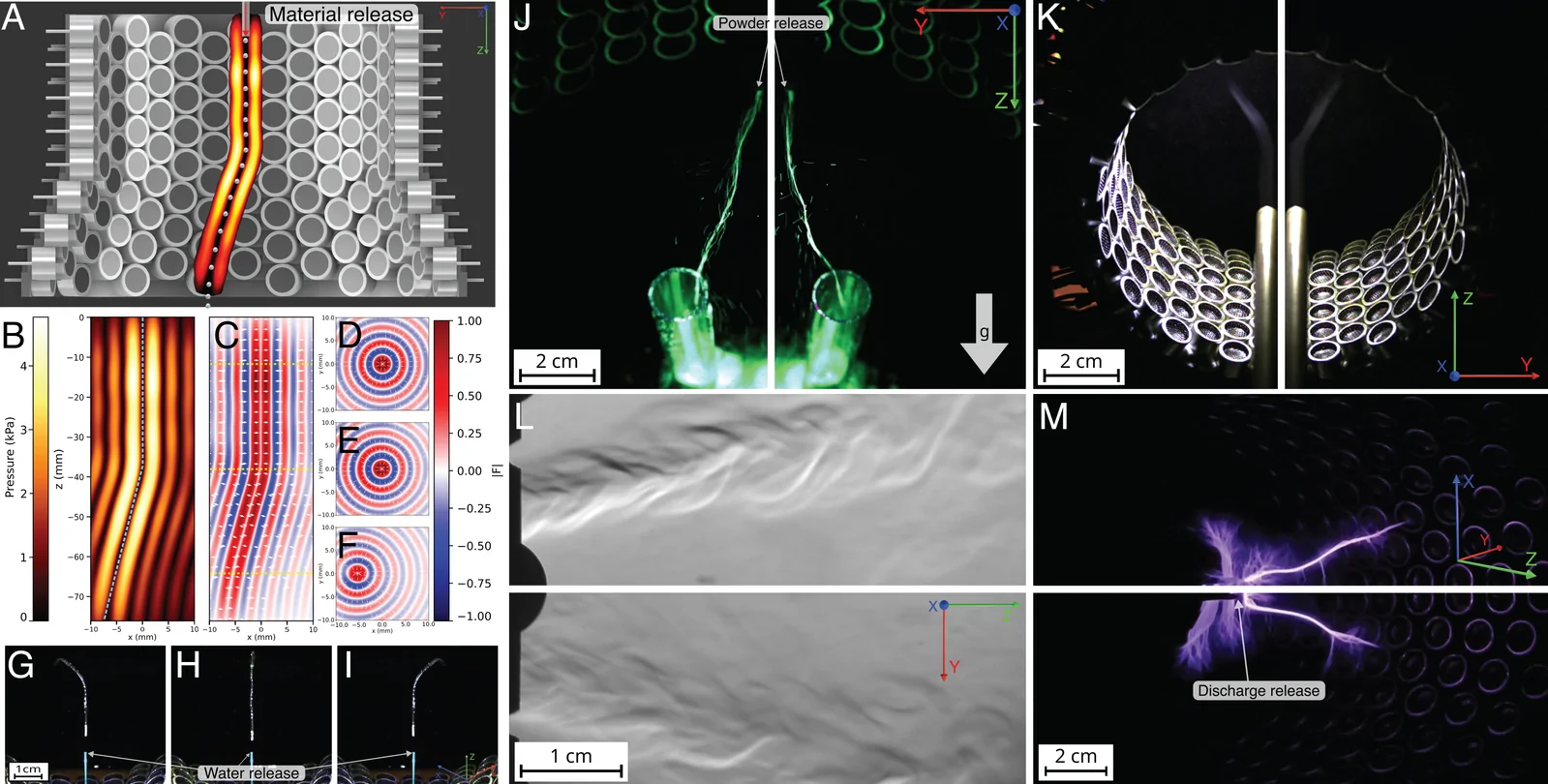
Guiding different states of matter using EBAFs generated by PATs. (*A*) Cross-section of the acoustic pressure isosurface of EBAF of mode *m* = 1 together with the PAT used for its generation. (*B*) Simulated acoustic pressure distribution of the EBAF of mode *m* = 1. (*C*) Corresponding ARF distribution. (*D*–*F*) Transverse distributions of ARF across different cross-sectional planes along the EBAF trajectory. (*G*–*I*) Experimental demonstration of horizontal water stream guidance using left-tilted, straight, and right-tilted EBAFs, respectively. (*J*) Composite image of polyethylene powder guided into two separate containers. *Left* and *Right* frames were captured at the moments of applying left- and right-tilted EBAFs. (*K*) Similar demonstration for water aerosol in horizontal EBAFs orientation. (*L*) Composite schlieren image illustrating upward and downward steering of an N_2_O gas stream. (*M*) Control of the direction of an electric discharge spark in a vertical plane.

An analytical method was developed (*SI Appendix*) to compute the drive signals required to synthesize EBAFs of specific radial distribution (modes) and trajectories (Fig. 1 *A* and *B* show the mode *m* = 1). EBAFs were generated using a custom-built PAT system, in which the transducers were mounted on connected cylindrical and conical surfaces (Fig. 1*A*). The acoustic radiation force (ARF) forms a channel-like region along the desired trajectory, confining particles within it (Fig. 1*B*–*F*) and enabling their guidance from the input point to the target location.

The length and path of EBAFs were dynamically reconfigured by adjusting the drive signals applied to the PATs, permitting on-the-fly adjustment of the guidance trajectory of matter. The resulting acoustic radiation force (ARF) was sufficiently strong to guide in a stable fashion a continuous stream of water along a horizontally oriented trajectory (Fig. 1*G*–*I* and Movie S3). For each material type, an appropriate EBAF configuration was selected to ensure stable guidance. This approach allowed guidance of liquids (Fig. 1*G*–*I*), powders and solid particles (Fig. 1*J*), aerosols (Fig. 1*K*), gases with acoustic properties different from those of air (Fig. 1*L*), and ionized gases (Fig. 1*M*).

### Different Modes of EBAFs.

**Fig. 2.**
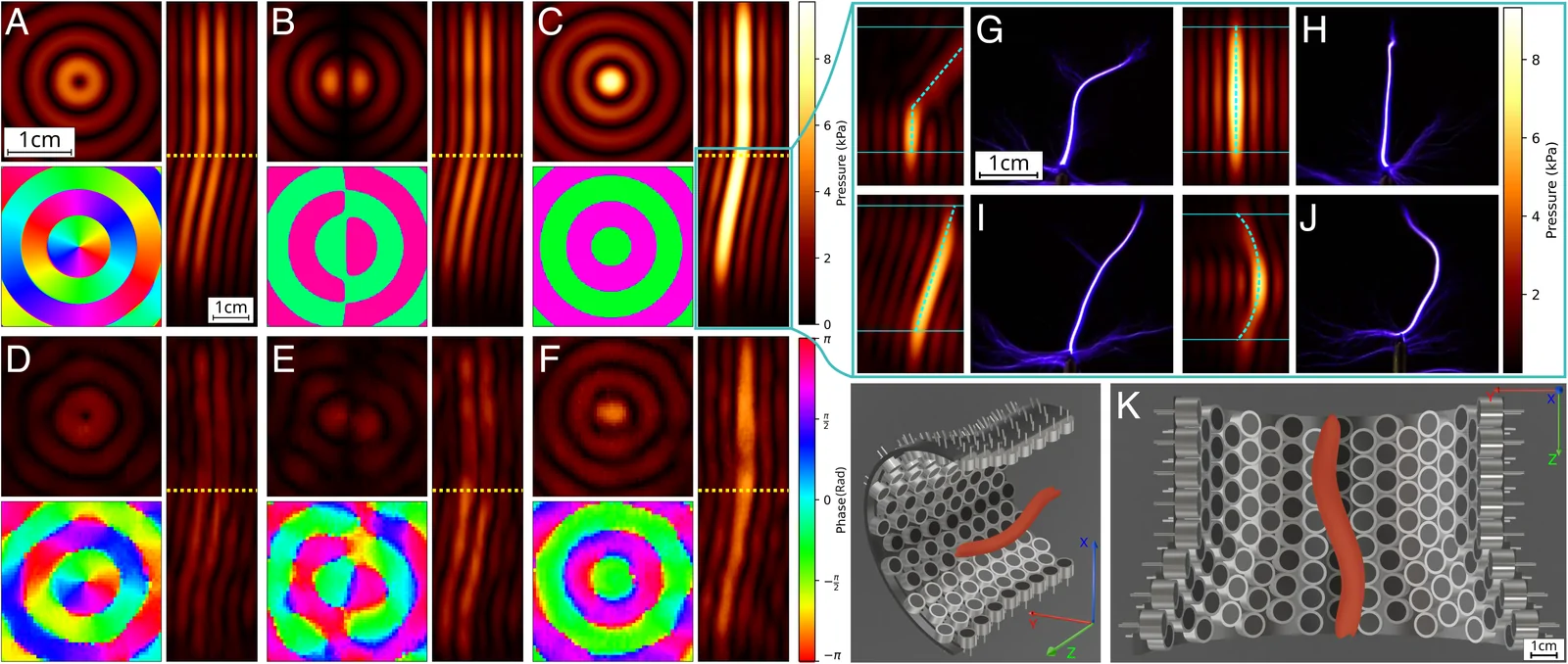
(*A*–*C*) Simulated EBAFs for modes *m* = 1, m=±1, and *m* = 0, respectively. For each case, the acoustic pressure amplitude in the axial plane is shown together with the transverse amplitude and phase distributions at the EBAF center. The corresponding maximum acoustic pressure amplitudes of these modes are 5.2 kPa, 5.3 kPa, and 9.2 kPa. (*D*–*F*) Corresponding experimentally measured acoustic fields obtained by microphone scanning with maximum acoustic pressure amplitudes of 3.1 kPa, 4.5 kPa, and 6.2 kPa. (*G*–*J*) Examples of *m* = 0 EBAFs of different trajectories generated using half of the PAT: (*G*) combination of straight and tilted segments, (*H*) straight field, (*I*) tilted field, and (*J*) half-period sinusoidal trajectory. The corresponding images of plasma guided along these trajectories are shown. (*K*) Simulated isosurface of an *m* = 1 EBAF following a 3D spiral trajectory.

In the current work we employed two distinct families of EBAFs. The first family consists of Bessel fields of order *m*, where m=0,1,..., with a complex pressure distribution in a transverse plane given by $p∼J_{m}\left(k_{0}r\right)\exp\left(im\varphi \right)$, where $J_{m}\left(\cdot \right)$ is the Bessel function of the first kind of order *m*, $k_{0}$ is the acoustic wavenumber, *r* is the radial distance from the field axis, *φ* is the azimuthal angle. For *m* = 0, the field is centrally focused along the axis (Fig. 2*C*). In contrast, for *m* > 0, the field forms a donut-shaped amplitude profile with a central minimum and an azimuthal phase that wraps *m* times around the circumference (Fig. 2*A* and *SI Appendix*, Fig. S3 *B*1, *D*1,and *F*1).

The second family denoted m=±1,m=±2,⋯, is formed by superposing two counterrotating Bessel fields of order +m and −m, resulting in the pressure distribution of $p∼J_{m}\left(k_{0}r\right)\cos\left(m\phi \right)$. These fields form 2m petal-shaped lobes separated by *m* nodal planes (Fig. 2*B* and *SI Appendix*, Fig. S3 *C*1, *E*1, and *G*1), with the acoustic phase remaining spatially constant within each lobe.

### Experimental Validation of EBAFs Generation.

To experimentally validate the proposed method for EBAF generation, the PAT was driven to produce specific EBAF configurations. The resulting fields were scanned using a calibrated microphone. Representative 2D field slices are shown in Fig. 2*D*–*F* (for the 3D field distribution, see Movie S2). Furthermore, the pressure amplitude and directivity pattern of an individual transducer were experimentally measured (*SI Appendix*, Fig. S2 *D*–*F*) and used as input for numerical simulations of the field generated by PAT (Figs. 1 *A* and *B* and 2*A*–*C*).

### Trajectory Control of EBAFs.

The proposed field generation method enabled the creation of EBAFs that follow predefined trajectories. Fig. 2*G*–*J* and Movie S4 show several examples of EBAF trajectories with mode *m* = 0, generated using the conical section of the PAT, along which an electric discharge was successfully guided. Moreover, the method supports the synthesis of more complex field geometries with variable lengths, limited only by the physical aperture of the PAT, as demonstrated by the numerically simulated 3D spiral trajectory shown in Fig. 2*K* and simulated EBAFs of different length and curvature shown in Movie S1.

### Guiding Particles in Different Modes of EBAFs.

**Fig. 3.**
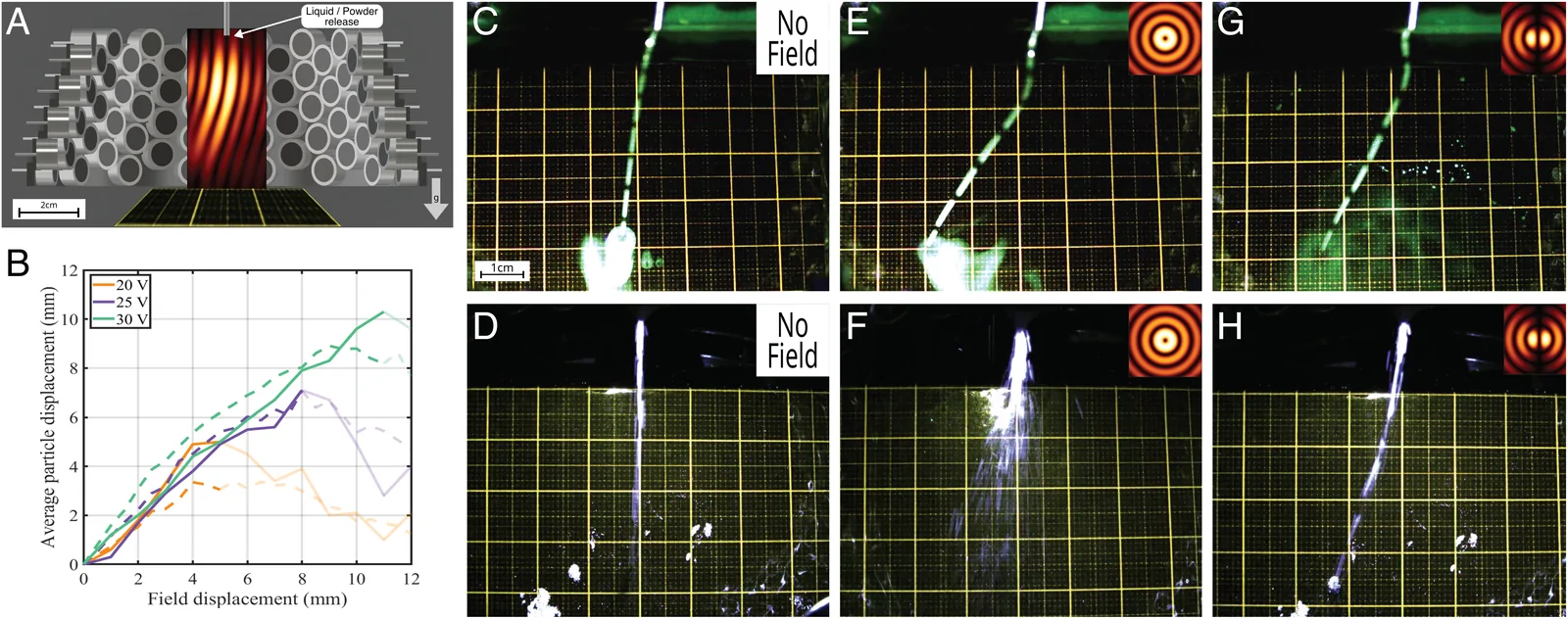
Ultrasonic guiding of water droplets and silica powder. (*A*) EBAFs were generated by the conical section of the PAT, forming a parabolic trajectory with a defined field displacement at the end of the PAT. The modes *m* = 1 and m=±1 were tested. (*B*) Results of silica particle displacement in EBAF of mode m=±1 over a displacement range from 0 to 15 mm, for the fields corresponding to 100%, 83%, and 67% of the maximum voltage of electrical signal ($U_{\mathrm{pp}}=30$ V). The solid and dashed lines represent the experimental and simulation results, respectively. Images showing water droplets and silica powder, respectively with no applied field (*C* and *D*), guided by the *m* = 1 mode (*E* and *F*), and by the m=±1 mode (*G* and *H*) with a field displacement of 15 mm.

To assess the matter guiding capabilities of different EBAF modes, we tested their ability to guide colored water droplets with diameter of [ to range-phrase = –, range-units = single]12, and silica powder with a mean particle diameter of 0.1 mm. The water droplets were released into the field through a needle, and the powder was introduced via a nozzle (Fig. 3*A* and Movie S3).

For water droplets, the modes *m* = 1 and m=±1 enabled transport from the ejection needle to the designated target point at the end of the PAT, as shown in Fig. 3 *E* and *G*, respectively. Notably, the *m* = 1 mode exhibited stronger particle retention along the trajectory compared to the m=±1 mode, as evidenced by the larger lateral displacement of droplets observed on the paper grid for the same field trajectory with a field displacement of 15 mm at the PAT exit. For the *m* = 1 mode, the maximum lateral displacement was equal to 15 mm. In comparison, the continuous liquid stream achieved a maximum lateral displacement of 16 mm (Movie S4). In both tests, the liquid was completely retained along the trajectory without any particle loss. Further quantitative assessment of material guidance is provided in *SI Appendix*.

In contrast, smaller particles (silica powder) could not be guided by the *m* = 1 mode (Fig. 3*F* and Movie S3), they exhibited a pronounced circumferential motion within the field, causing them to escape the EBAF. The *m* = 0 mode could not guide these particles either, as they were pushed away from the central field focus, resulting in uncontrolled off-axis trajectories. On the other hand, the mode m=±1 provided effective confinement and guidance of these fine particles along the predefined path (Fig. 3*H* and Movie S3). Under m=±1 mode excitation, the powder stream achieved a maximum lateral displacement of 10 mm, with a maximum path deviation of 2 mm relative to the intended displacement. The powder stream exhibited only minor fallout of grains along the trajectory, representing a negligible fraction relative to the total guided mass.

### Particle Motion Simulation in EBAF.

A model using a superposition of the radiated fields from individual transducer (piston) sources was developed to simulate particle motion in the EBAF, incorporating ARF, Stokes drag, and gravity. To experimentally validate the model, parabolic EBAFs with varying lateral displacements at the PAT end were generated. The resulting particle displacements were measured and compared with the simulation results for different acoustic pressure levels over a field displacement range from 0 to 15 mm. These results highlight the importance of selecting appropriate pressure levels to guide particles to the intended target, with higher acoustic pressures required to displace particles farther from the vertical axis. As shown in Fig. 3*B*, the simulations predicted particle dropping from the EBAF and average displacement at a target plane, and can be used to predict particle motion within the EBAF.

A second FEM simulation investigated the observed particle ejection from the EBAF mode *m* = 1 (*SI Appendix*, Fig. S8). The model agreed with the observed ejection of powders, aerosols, and gases. The observed orbital motion of particles was attributed to the drag force induced by acoustic streaming. The simulation further revealed that the guiding capability of the *m* = 1 EBAF strongly depends on particle size: simulated silica particles with a diameter above 150 to 200 μm were ejected from the EBAF axis. In contrast, the m=±1 mode did not exhibit a strong size dependence. These results show that the balance between acoustic radiation forces and acoustic streaming induced drag determines the boundary between stable guidance and particle ejection. The present numerical model therefore provides a basis for estimating this boundary for a given material and acoustic-field configuration.

### Gases, Gaseous, and Ionized Media Guiding.

**Fig. 4.**
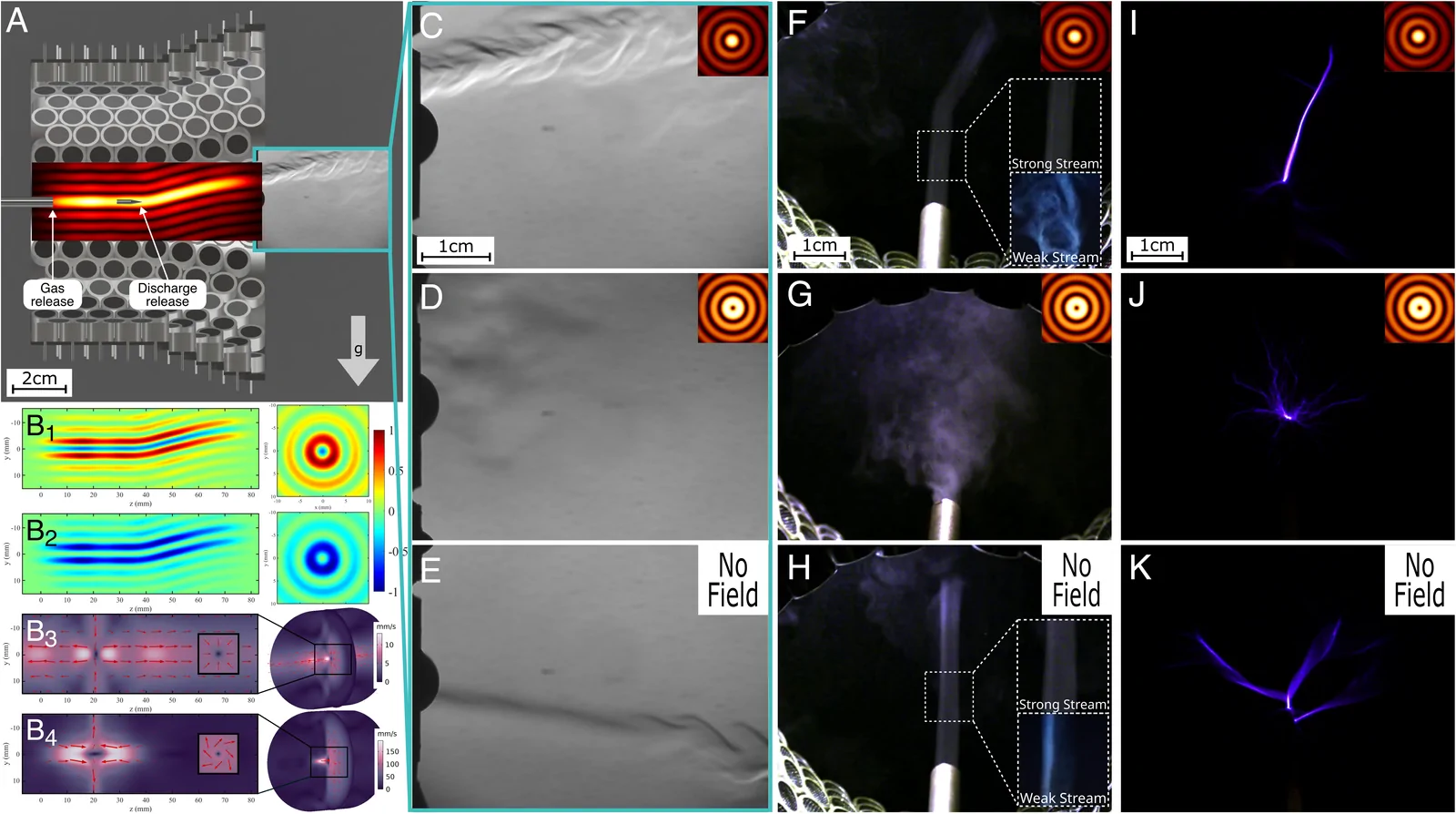
Ultrasonic guiding of N_2_O gas, water aerosol, and hot ionized air produced by a Tesla coil. (*A*) The generated EBAF consisted of a straight segment formed by the cylindrical section of the PAT and a tilted straight segment formed by its conical section, producing a defined lateral offset at the *Right* side of the PAT. The gas and aerosol were injected at the PAT *Left* side, while the discharge needle was positioned at the midpoint of the array. (*B*1 and *B*2) Gor’kov potential energy distribution for N_2_O gas and hot air at 70 ^°^C, respectively. (*B*3 and *B*4) Simulated acoustic streaming patterns for modes *m* = 0 and *m* = 1 generated by the cylindrical section of the PAT. (*C*–*E*) Schlieren images of N_2_O gas near the PAT *Right* side. The behavior of CO_2_ gas is qualitatively identical. (*F*–*H*) Camera images of guided water aerosol. (*I*–*K*) Representative images from Movie S4 of Tesla coil electric discharge in air. For each group (*C*–*K*), the first row corresponds to mode *m* = 0, the second to mode *m* = 1, and the third to the case without an applied acoustic field.

Different EBAFs modes were tested for guiding gases and gas-like media, including N_2_O and CO_2_ gases, water aerosol, and ionized air generated by a Tesla coil discharge. Effective guidance of these media was achieved with the *m* = 0 mode with the full-length array enabled (Fig. 4*C*, *F*, and *I* and Movie S4) and with half of the array length enabled (Movie S6, *Right* panel).

N_2_O, CO_2_, and water aerosol were introduced into the EBAF through a pipe aligned with the field origin (Fig. 4*A*). The media exhibited stable flow along the field trajectory (Fig. 4 *C* and *F* and Movie S4). The mode *m* = 1 produced a turbulent flow pattern and failed to maintain stable guidance along the trajectory defined by the EBAF (Fig. 4 *D* and *G* and Movie S4). The mode m=±1 provided only partial confinement, as the media tended to escape through the lateral pressure minima of the field, as can be seen in Movie S4.

In the experiment with ionized air, the metal discharge needle was placed at the center of the PAT, where the EBAF begins to turn (Fig. 4*A*). Before activating the field, the Tesla coil produced randomly directed discharges in the air. Once the field was activated, the discharge stably followed the EBAF trajectory (Fig. 4*I*).

Analysis of the discharge trajectory showed that, under the *m* = 0 EBAF, the maximum lateral deviation of the discharge from its stable position within the EBAF was approximately 1 mm, comparable to the characteristic diameter of the visible part of the discharge channel, which was approximately 1 to 2 mm. In contrast, under field-off conditions, the discharge paths were stochastic and exhibited lateral deviations of up to 25 mm. Other EBAF modes failed to provide stable guidance of the discharge. In particular, the *m* = 1 mode produced stochastic trajectories with transverse deviations of approximately 15 mm (Fig. 4*J*).

### Applications.

**Fig. 5.**
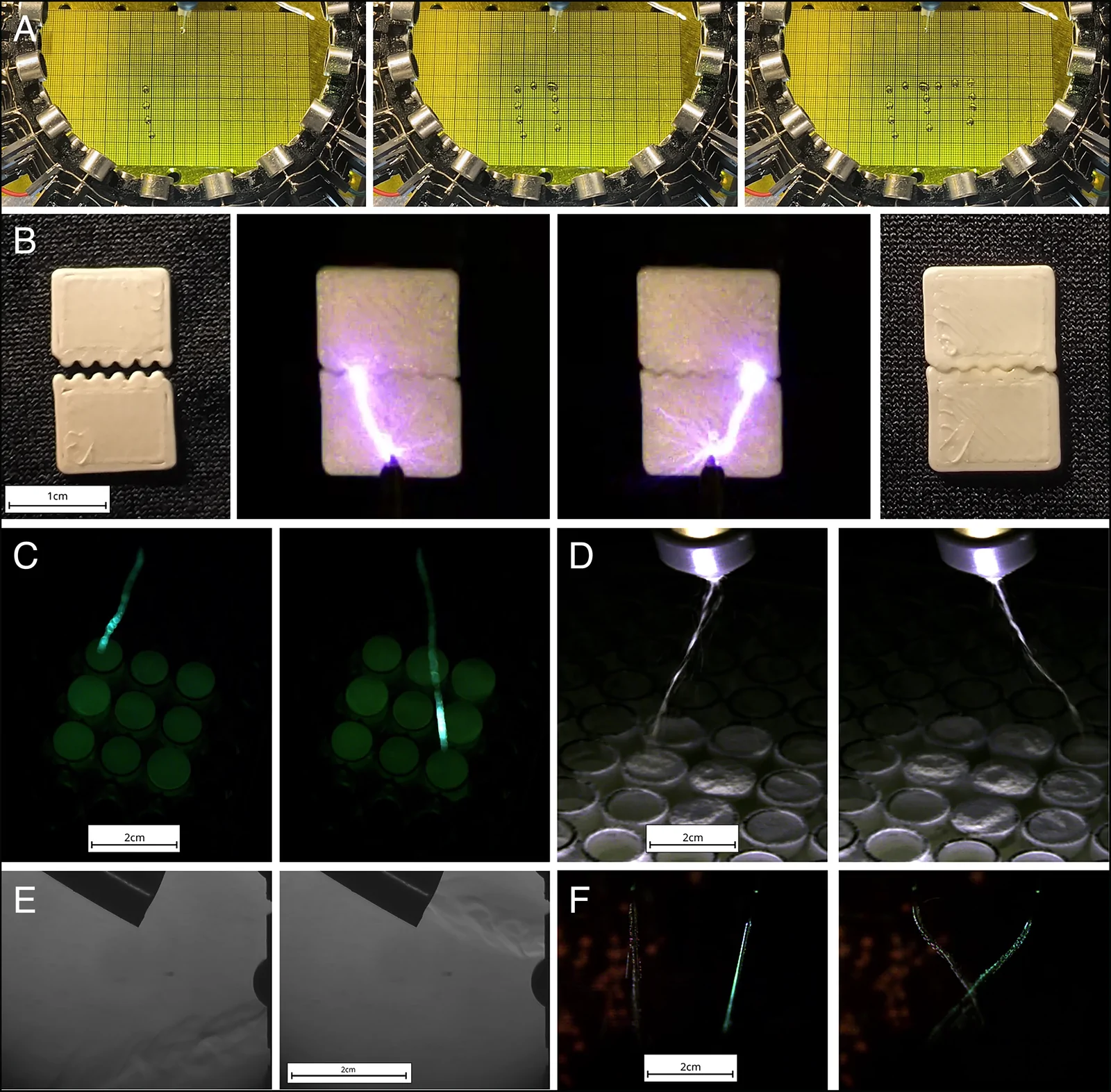
Potential applications of EBAFs. (*A*) Time-lapse showing controlled deposition of liquid droplets onto a substrate to form the letter “E.” (*B*) Continuous steering of an electric discharge along the edges of two separate PLA plastic pieces for precision welding. (*C* and *D*) Steering of liquid and powder streams for targeted filling of a well-plate. (*E*) Controlled capture of N_2_O gas. (*F*) In-air mixing of two liquid streams. See Movie S5 for recordings of these processes.

The ability to control laminar gas flow opens up opportunities for targeted capture of gases and aerosols (Fig. 5*E*) and for directing them toward processing areas e.g., atmospheric pressure chemical vapor deposition. Another promising use is the steering of ionized gas along an intended trajectory, enabling noncontact welding (Fig. 5*B*). See Movie S5 for the recordings of these experiments.

## Discussion

### Mechanisms of Interaction of EBAFs with Different Types of Matter.

The interaction of particles with EBAFs is governed by two physical mechanisms. The first is ARF, which depends on the spatial distribution and magnitude of the acoustic pressure and the acoustic contrast factor (ACF) between the particle and the surrounding medium (52). Particles with positive ACF are driven toward pressure nodes, while those with negative ACF move toward antinodes. The mode *m* = 0 was suitable for guiding both ionized hot air with a negative ACF in the central pressure maxima, and heavy gases with a positive ACF, which were guided in a tubular region surrounding the central pressure maximum. The modes m=1,±1,2,±2,⋯ were effective for solid particles and droplets which have a positive ACF.

The second mechanism is the drag force due to acoustic streaming, which becomes particularly pronounced for modes m=1,2,…. In these modes, the streaming flow drives orbital particle motion around the EBAF axis (53), which can ultimately eject particles from the field center.

The ratio between ARF and drag force strongly depends on particle size and thus defines the particle behavior. Smaller particles are captured more rapidly by fluid flow due to their shorter Stokes relaxation time $\tau =\frac{\rho_{p}d_{p}^{2}}{18\mu }$ (54), where $\rho_{p}$ is the particle density, $d_{p}$ is the particle diameter, *μ* is the dynamic viscosity of air. The relaxation time of the powder particles used in this work was *τ* ≈ 110 ms, while for liquid droplets it was *τ* ≈ 7 s. Given that the characteristic free-fall time over the PAT length is ∼0.1 s, the powder particles have sufficient time to reach streaming velocities during their descent. In contrast, droplets cannot accelerate to such velocities within the guiding time. As a result, water droplets remain stably trapped by the ARF near the EBAF center, whereas smaller powder particles are entrained by streaming and eventually expelled once centrifugal forces exceed the ARF (*SI Appendix*, Fig. S8).

### Effects on Gases and Gaseous Media.

Observations in aerosol experiments revealed that the aerosol particles were displaced from the central pressure maximum toward a surrounding pressure minimum, forming a cylindrical shell along which they propagated. This behavior can be explained by the positive ACF of the aerosol particles, which drives them toward regions of minimum acoustic pressure. However, carrier-flow entrainment may also contribute to the observed guidance. The gas exiting the humidifier is cooler than the surrounding ambient air due to the endothermic evaporation of water droplets, and as shown in Movie S7, the cold gas flow can itself be guided by EBAF. Thus, in the mist experiment, direct acoustic interaction with the droplets and carrier-flow entrainment may act simultaneously and in the same direction, contributing to the observed guidance of the mist.

The inability of the *m* = 1 mode to guide gaseous media, as in the powder experiments, is attributed to strong circumferential acoustic streaming (*SI Appendix*, Fig. S7). Although the gas and aerosol streams exiting the injection pipe were initially laminar (*SI Appendix*, Fig. S7*E*), experimental observations showed that any transition to turbulence disrupted acoustic guidance. Effective guidance therefore requires minimizing both acoustic streaming and ambient airflow disturbances. In the experiments, ambient airflow disturbances were minimized by enclosing the device with fabric. The effect of acoustic streaming was minimal for the mode *m* = 0, for which simulations revealed no rotational component of the streaming field and a maximum streaming velocity 15 times lower than that of *m* = 1 (*SI Appendix*, Fig. S7 *C*1 and *C*2), thereby excluding gas mixing and enabling effective guidance.

The m=±1 mode also appears suitable for guiding aerosols and gases, as it features a localized low-potential region along the axis and generates streaming velocities significantly lower than those of the *m* = 1 mode (*SI Appendix*, Fig. S7 *C*2 and *C*3). In experiments, the aerosol stream was compressed into an oval-shaped profile, which can be explained by the streaming pattern directed toward the axis squeezing the aerosol stream from the sides (*SI Appendix*, Fig. S7*C*3). However, the relatively strong streaming velocities were sufficient to disturb the flow near the end of the field. Overall, the guiding efficiency of the m=±1 mode was lower than that of *m* = 0 but higher than that of *m* = 1.

Taken together, the experiments and simulations detailed in *SI Appendix* indicate that the guidance of gaseous media is strongly governed by the acoustic properties of the gas relative to the surrounding air. For the *m* = 0 EBAF, denser than air gases exhibited stable confinement and followed dynamically reconfigured trajectories, whereas the investigated lighter than air gases could not be stably guided under the tested conditions.

Finally, control experiments detailed in *SI Appendix* and Movie S6 confirm that acoustic streaming exerts no significant influence on gas guidance for mode *m* = 0, proving that ARF, associated with acoustic contrast, is the primary physical mechanism governing gas routing.

### Acoustic Guidance of Ionized Media.

The Gor’kov potential energy distributions for the heavy N_2_O gas and for the localized hot region associated with the electric discharge (55) are noticeably different, as shown in Fig. 4*B*1 and *B*2. In both cases, the guided material tends to occupy regions of low acoustic potential energy. For heavy gases, including CO_2_ and N_2_O, as well as water aerosol, this corresponds to a tubular region surrounding the central pressure maximum. This behavior is supported by the experimental observations, where the aerosol forms a hollow, tube-like structure under slow-flow conditions (Fig. 4*F*). In contrast, the electric discharge follows the central axis of the EBAF, consistent with the corresponding acoustic potential-energy distribution and the experimental observations.

The different behavior of the discharge compared with the bulk hot-air stream, which was not guided by the EBAF, can be understood from the substantially different spatial scales of the two thermal perturbations. While the bulk heated-air stream used in the control experiments (Movie S6) was relatively broad and did not exhibit stable guidance by the *m* = 0 EBAF, the electric discharge produces a highly localized and continuously heated region. Recent work by Irisarri et al. (55) showed that such localized thermal perturbations can experience strong acoustic interaction and become confined by an acoustic pressure antinode. Thus, we argue that gas and discharge guidance can be described by the same underlying ARF mechanism. However, the observation that the discharge follows the EBAF axis does not necessarily imply that a bulk hot-air stream should be similarly guided. The highly localized nature of the discharge and its continuously maintained thermal perturbation may result in substantially stronger acoustic confinement than that of the broader hot-air stream.

Following the potential energy interpretation, the mode *m* = 1 should, in principle, guide heavy gases along its central pressure node, since it exhibits a minimum of potential energy along the field axis (*SI Appendix*, Fig. S7*B*2). In practice, however, the experiments revealed turbulent flow for *m* = 1 (Fig. 4*G*). This discrepancy was attributed to the generation of strong acoustic streaming. FEM simulation (Fig. 4*B*4) confirmed that the mode *m* = 1 produced the strongest acoustic streaming among all tested modes, with maximum velocity approaching 200 mms^−1^ with a pronounced rotational component. This high-speed vortex flow disrupted the otherwise directed transport of gas and aerosol, promoting mixing with the surrounding air and preventing stable guidance along the potential-energy minimum.

### Field Simulation and the Effect of the Reflections.

The simulated fields were calculated as a superposition of the direct waves radiated by the PAT transducers. As shown in *SI Appendix*, Fig. S2 *A* and *B*, the resulting spatial distribution is uniform, and the field magnitude scales proportionally with the pressure amplitude of a single transducer. However, experimental measurements for the *m* = 1 mode (*SI Appendix*, Fig. S2*C*) revealed a significant reduction in field uniformity and a pronounced drop of amplitude at 40 kHz despite this being the nominal operating frequency of the transducers. In contrast, the *m* = 0 mode displayed a different behavior, with the pressure magnitude closely matching the pressure amplitude of a single transducer profile (*SI Appendix*, Fig. S2*D*).

The discrepancy between the simulated and experimental fields arises from acoustic reflections inside the closed cylindrical geometry of the PAT surface. As a result, the effective operational frequency of the PAT must be determined not only by the transducer amplitude frequency response but also by the resonance conditions of the array (56). In the present experiments, 40 kHz was chosen for the *m* = 0 mode, whereas 39 kHz was used for the *m* = 1 and m=±1 modes to maximize the acoustic pressure amplitude of each respective EBAF thereby providing the strongest material retention and the highest degree of control over material routing.

As shown in *SI Appendix*, Fig. S6, the conical section produced acoustic fields that matched the simulated fields more closely than the cylindrical section, particularly in the region where the EBAF trajectory was tilted. This improvement is attributed to the expanding geometry, which allows reflected acoustic waves to propagate out of the active control region, thereby reducing their interference with the generated acoustic field. Therefore, the conical geometry was used in the trajectory control region throughout all experiments.

### Throughput and Potential Applications.

The demonstrated material delivery rates are relevant to several potential additive manufacturing applications. For powder-fed laser directed energy deposition (DED), reported powder delivery rates are typically approximately 5 to 20 g/min (57). The zirconia/silica powder used in the present study was delivered at 4.2 g/min, which is close to the lower end of this reported range. Similarly, extrusion-based bioprinting commonly operates over a broad range of material flow rates, approximately 0.1 to 10 mL/min, depending on bioink properties, nozzle geometry, and processing conditions (58). The continuous liquid streams in our experiments were guided at 10 to 30 mL/min. Although these comparisons do not represent direct demonstrations of DED or bioprinting, they indicate that the demonstrated acoustic material delivery rates are relevant to existing printing regimes.

The maximum achievable throughput is expected to depend strongly on the EBAF trajectory curvature, acoustic pressure amplitude, material extraction conditions, initial material velocity, and material flow rate. These parameters can potentially be optimized for a specific application to achieve the required balance between material throughput and stable guidance.

### EBAFs in Liquid Media.

This approach can be extended to operate in liquids. The magnitude of ARF scales as $F_{\text{rad}}\propto \frac{P^{2}k_{0}}{\rho c^{2}}$, where *P* is the acoustic pressure amplitude, $k_{0}$ is the wavenumber, *ρ* and *c* are the medium density and speed of sound, respectively. Achieving the same ARF in water requires the pressure amplitude to be two orders of magnitude higher and the frequency one order of magnitude higher. In this work, an amplitude of 5 and a frequency of 40 were used. To achieve a comparable ARF in water, the amplitude should reach the MPa range, and the frequency should be 0.1, both requirements are achievable with existing underwater PATs. To date, experimental acoustic guidance of specific materials, such as bubbles, along arbitrary trajectories in liquid media has been demonstrated (42). The same principle could potentially be extended to other materials in liquids.

### Conclusion.

A family of acoustic fields, Elongated Bessel Acoustic Fields (EBAFs), was proposed, together with a practical method to generate them along arbitrary paths using a phased array of transducers. The proposed generation technique was experimentally validated, confirming the feasibility of producing EBAFs with dynamically reconfigurable trajectories.

EBAFs enabled continuous contactless guidance of liquids, solids, gases, and ionized gases, across a broad range of particle sizes, from aerosols and powders to millimeter-scale particles, as confirmed through a series of experimental demonstrations. The underlying physical mechanisms governing the behavior of each type of medium in EBAFs were identified, primarily involving acoustic radiation and drag forces. Understanding these mechanisms allowed the selection of suitable EBAF modes optimized for different materials and particle sizes.

Several proof-of-concept demonstrations highlight the versatility of using EBAFs, including controllable droplet deposition, dosing of liquids and powders, mixing of reagent streams, targeted capture of gases and aerosols, and noncontact welding.

## Materials and Methods

### Phased Arrays of Transducers–PATs.

EBAFs were generated using a custom-built PAT system (Fig. 1*A* and *SI Appendix*, Fig. S1), driven by a multichannel signal generator capable of independently controlling the amplitude and phase of each emitter. PATs were assembled in an axisymmetric layout and consisted of two segments, each with 128 emitters. The housing for the transducers was 3D printed from PLA plastic on an Ultimaker 3 FDM printer (Ultimaker B.V., Netherlands). The emitters (MSO-SAZ1040DH07T, Manorshi, China) with a diameter of 9.8 and a central frequency of 40 were inserted inside the printed housing. The dimensions of the cylindrical segments were 89 in diameter and 40 in length and the diameters of the conical segment were 89 and 119, the length was 40 (Fig. 1*A*). The transducers were arranged with an axial pitch of 5.08 mm and 16 transducers distributed circumferentially around the array. We note that other PATs geometries can be assembled from cylindrical and conical segments, e.g. two cylindrical and a conical segment.

PATs were driven by three FPGAs (XC6SLX16, Numato Lab, India), each controlling 120 channels. The FPGA generated a square wave signal for each channel, which was amplified up to 30 by a 10-channel level shifter integrated circuit (TPS65190, Texas Instruments, USA). The phase of each channel was controlled by introducing a time delay relative to a shared FPGA clock. The driving system enabled independent control of phase, frequency, and duty cycle for each transducer with resolutions of π/50, 10 and 1%, respectively. Due to the filtering properties of the transducers, the resulting emitted signal is a continuous sine wave. For the detailed description of the driving electronics see ref. 59.

### Acoustic Field Scanning.

The acoustic pressure field was recorded using a microphone (46DE 1/8” CCP Pressure Microphone, GRAS, Denmark) connected to a digital oscilloscope (PicoScope 3403D, Pico Technology, United Kingdom). The microphone was mounted on a linear XYZ translation stage (T-type Sliding Stage, Toseastars, China) driven by a stepper motor (Nema 11 Stepper Motor, Toseastars, China). A custom-built software package implemented in Python controlled the translation system, defined the scanning parameters (including scan plane, area, and step size), acquired acoustic signals, and converted them into amplitude and phase representation.

### Imaging of the Experiments.

The imaging of gas flow was done with a schlieren setup, the detailed description of which is provided in ref. 60. Imaging in other experiments was done with a camera (DFK 23UP1300, Imaging Source, Germany) fitted with a lens (8 to 50 mm Zoom Lens for Pi, Waveshare, China).

### Materials and Operating Conditions for Acoustic Guiding.

The solid particles used in the experiments included zirconia/silica microspheres (BioSpec Products Inc., Bartlesville, OK, USA) with a particle diameter of 100 μm and a density of 3.7 g/cm^3^; fluorescent green polyethylene microspheres with a diameter of 100 μm and a density of 1.0 g/cm^3^ (Cospheric LLC, California, USA); and plastic beads with a diameter of 0.8 mm and a density of 2.18 g/cm^3^ purchased from a local store. The particles were released into the operating area through a 3D-printed nozzle, with a 0.8 mm opening for powders and a 2 mm opening for plastic beads (*SI Appendix*, Fig. S1). The particle release rates were 4.2 g/min, 1.4 g/min, and 20 g/min for zirconia/silica, polyethylene, and plastic particles, respectively. The initial velocity of the released materials was approximately zero.

For the liquid experiments, either pure water or water dyed with Rhodamine B (RhB) for fluorescence imaging was used. The liquid was injected into the operating area using a syringe pump (Nanojet, Chemyx, Stafford, USA) connected to a needle 21G (*SI Appendix*, Fig. S1). The pumping rate in these experiments varied between 10 to 30 mL/min. The experiments were performed at room temperature, and the density of water under these conditions was approximately 998 kg/m^3^.

Aerosol was produced using a portable humidifier, and the mist was transferred to the operating area through a metal tube with an inner diameter of 5 mm. The aerosol droplet-size distribution was not independently characterized in the present study, the reported Sauter mean diameter for mist produced using the same method under standard conditions is equal to $d_{32}\approx 13\;\mu \text{m}$ (61). No external carrier gas was used in the aerosol experiments. The mist stream velocity was measured using shadowgraph technique and was equal to 0.42 m/s.

For the gas experiments, carbon dioxide CO_2_ ($\rho \approx 1.84\;{\text{m}}^{-1}$), nitrous oxide N_2_O ($\rho \approx 1.83\;{\text{m}}^{-1}$), and helium He ($\rho \approx 0.166\;{\text{m}}^{-1}$) were used. These gases were released from pressurized containers and directed into the operating area through the metal tube with an inner diameter of 5 mm (*SI Appendix*, Fig. S1). These gases were released from pressurized containers and directed into the operating area through the same metal tube (*SI Appendix*, Fig. S1), with an inner diameter of 5 mm. The room temperature gas flow rate was maintained at approximately 1 L/min measured with Mass Flow Meter GFM37 (Aalborg, USA) corresponding to a mean flow velocity of approximately 0.85 m/s at the tube outlet. The inlet pressure was not measured directly, as the gas flow rate was the controlled experimental parameter. The corresponding outlet flow velocity is therefore reported as the primary parameter characterizing the injected gas stream.

An electric discharge was generated using a Tesla coil (Yosoo Health Gear, China) operating at 2.4 MHz. The signal was modulated using a rectangular waveform at 2.5 kHz with 50% duty cycle. The Tesla coil was powered by a regulated DC supply with an input voltage of 48 V and an average input current of approximately 1 A. The absolute high-voltage output, discharge current, and discharge power at the electrode were not directly measured in the present study. The discharge occurred at the tip of a sharp-tipped metallic needle (*SI Appendix*, Fig. S1) positioned inside the PAT. The electrode was approximately 30 mm long and 2 mm in diameter, with a sharpened tip extending approximately 4 mm.

### Algorithm for Generation of EBAFs Shaped Along 3D Paths.

A mathematical method was developed for generating the desired EBAFs (*SI Appendix*). The corresponding algorithm for calculating drive signals was implemented in both MATLAB (R2023a, MathWorks, USA) and Python. It determines the required amplitude and phase of each transducer in the PAT based on the array geometry, field frequency, and the specified EBAF trajectory and mode. These parameters are then converted into duty cycles and time delays for the FPGA drive signals. A detailed description of the algorithm is provided in *SI Appendix*.

### Simulation of the Acoustic Pressure Field Generated by PAT.

The acoustic pressure field generated by the PAT was modeled as a superposition of the radiated fields from individual piston sources, each characterized by its experimentally determined radiation pressure amplitude and effective diameter. The simulation algorithm was implemented in both MATLAB (R2023a, MathWorks, USA) and Python. A detailed description of the algorithm is provided in *SI Appendix*.

### FEM Simulation of Acoustic Streaming and Particle Tracing.

FEM simulations were built with COMSOL Multiphysics^®^ 6.2 (COMSOL AB, Sweden). A 3D model was used to simulate the acoustic streaming patterns for different EBAF modes in the straight section of the PAT. A 2D simulation was used to study the effect of acoustic streaming on the particle trapping ability in different fields. In both models, the acoustic streaming field was calculated using the built-in coupling between the pressure acoustics in the frequency domain and the stationary laminar flow. In the 2D particle tracing simulation, the applied forces included Stokes’ drag, Saffman lift, and ARF. The Gor’kov formulation was used for ARF. Particles were initially released in a randomized grid within the central acoustic node with zero initial velocity.

For 3D particle tracing simulations ARF, air drag, and gravity were considered. Particles were released, as done in ref. 62, following a 2D spatial normal distribution with a SD of 0.4, corresponding to half the nozzle size, with a low initial downward speed of 20 mm/s and a normal sideways distribution with a SD of 15 mm/s.

## Supplementary Material

Appendix 01 (PDF)

Movie S1. This video summarizes the study, illustrating the principle of Elongated Bessel-like Acoustic Fields (EBAFs) generation using PATs, and the resultant continuous guidance of material in various states along three-dimensional paths.

Movie S2. 3D field distributions comparing experimental scans with numerical simulations of EBAFs for m=1 straight (0:00-0:31), m=1 tilted (0:31-1:02), m=2 straight (1:02-1:33), m=2 tilted (1:33-2:05). The trajectory curving pf EBAFs of the modes *m* = ±1, *m* = 1, and *m* = 0 is shown in 2:05-2:16.

Movie S3. Demonstration of the guidance of laminar liquid stream (0:00-0:34), droplets (0:34-0:53), powder (0:53-1:40), and solid particles (1:40-1:47) via different EBAF modes and identifies the optimal mode for each case.

Movie S4. Guidance of water aerosol (0:00-0:46), N_2_O (0:46-1:20), and ionized air generated using Tesla coil discharge (1:20-2:03), demonstrating that the optimal EBAF mode identified for aerosols provides effective guidance of all these gaseous media.

Movie S5. Demonstration of application scenarios enabled by acoustic guidance, including controlled material deposition (0:00-0:10), non-contact welding processes (0:10-0:24), gas capturing (0:24-0:35), directed liquid and powder transport (0:34-0:49), and liquids mixing (0:49-1:02).

Movie S6. 0:00 - 0:12 Comparison of the effect of the acoustic field on room-temperature air (20°C) and the heavy gas HFO-1234ze under identical acoustic conditions. 0:12 - 0:22 Comparison of the effect of the acoustic field on heated (200°C) air and the heavy gas HFO-1234ze under identical acoustic conditions.

Movie S7. Steering of CO_2_, N_2_O, cold air at 1^°^C, and He under identical *m* = 0 EBAF conditions, with the acoustic trajectory displaced by 10 mm in both upward and downward directions.

Movie S8. FEM simulations of the dynamics of gases with different acoustic properties under identical *m* = 0 EBAF conditions. The upper panels show the temporal evolution of gas density and flow velocity for gases lighter than air (H_2_, He, CH_4_, and air at 200^°^C) and gases denser than air (air at 1^°^C, N_2_O, CO_2_, and Xe). The simulations demonstrate stable confinement and restoring motion toward the EBAF axis for the investigated denser than air gases, whereas the lighter than air gases progressively move away from the central region. For comparison, the movie also shows the evolution of He in the absence of the acoustic field, where only diffusion is considered, together with the corresponding simulation with the acoustic field. The movie also shows the calculated normalized acoustic radiation pressure at a sharp gas-air interface as a function of gas density and sound speed. The map illustrates the dependence of the ARF direction on the combined acoustic properties of the gas and surrounding air. The locations of the investigated gases are indicated, showing correspondence between the radiation-pressure regions and the experimentally observed guidance and no-guidance behavior. For further details of the radiation-pressure map and its interpretation, see the section “FEM simulation of gas dynamics.”

## Data Availability

The code supporting the findings of this study are publicly available in Zenodo at https://doi.org/10.5281/zenodo.23112817 (63). The repository contains the codes supporting the results presented in this article. Algorithms and theoretical derivations are included in the article and *SI Appendix*.
