# Supplementary material for: Contactless continuous guidance of matter in its main states using curved elongated Bessel-like acoustic fields
Source: Proc Natl Acad Sci U S A. 2026 Oct 23;123(44):e2614959123. doi: 10.1073/pnas.2614959123 (PMC13732203; doi:10.1073/pnas.2614959123)
Supplement: Supplementary file 1 [file pnas.2614959123.sapp.pdf]

Supporting Information for

Contactless Continuous Guidance of Matter in its Main States using Curved Elongated Bessel-like Acoustic Fields

Dmitrii Nikolaev, Denys Iablonskyi, Alexander Martinez-Marchese, Marika Sirkka, Mikel Aldea, Chinedum Okwudire, Edward Hæggström, Asier Marzo, Ari Salmi

Dmitrii Nikolaev.
E-mail: dmitry.nikolaev@helsinki.fi

This PDF file includes:

Supporting text
Figs. S1 to S8
Table S1
Legends for Movies S1 to S8
SI References

Other supporting materials for this manuscript include the following:

Movies S1 to S8

Supporting Information Text

Experimental setup

Figure S1 presents the experimental configuration for field scanning and matter ejection in the operation region, followed by subsequent guiding using the field generated using PAT.

EBAF generation and real-time update. The electrical drive-signal sets for all transducers required to generate the desired EBAF trajectories were calculated in advance using the EBAF generation algorithm implemented in MATLAB R2023a (MathWorks, USA). The calculation of a complete set of drive signals typically required approximately 1 s on a Lenovo ThinkPad T14 equipped with an 11th Gen Intel Core i5 processor and 16 GB of RAM. A maximum of 31 trajectories were used in this work, corresponding to a total calculation time of less than a minute. The resulting signal sets were uploaded to the FPGA RAM before the experiments. During operation, predefined signal sets could be switched with a minimum interval of 20 ms, with the drive signals for all transducer channels updated simultaneously. This enabled real-time reconfiguration of the EBAF trajectory without recalculating the drive signals during operation. In the experiments presented in this work, trajectory configurations were typically switched at intervals of 200 ms to 1 s.

The physical formation of the updated acoustic field is additionally determined by the acoustic propagation time. At the array elements, the updated phase and amplitude configuration is established after a short transient of approximately 20 acoustic periods, determined by the bandwidth of the transducers, corresponding to approximately 500 μs at the acoustic frequency of 40 kHz.

The acoustic field then propagates from the transducers to the points along the EBAF trajectory. For a maximum propagation distance in the array of approximately 10 cm, the corresponding acoustic time of flight is approximately 0.29 ms, assuming a sound speed of 343 ms^{-1}. Thus, the acoustic propagation time along the EBAF trajectory is on a submillisecond timescale, substantially shorter than the trajectory switching intervals used in the experiments.

Workflow of the EBAF generation. Here, we provide the workflow used to generate the transducer drive signals from a prescribed EBAF trajectory.

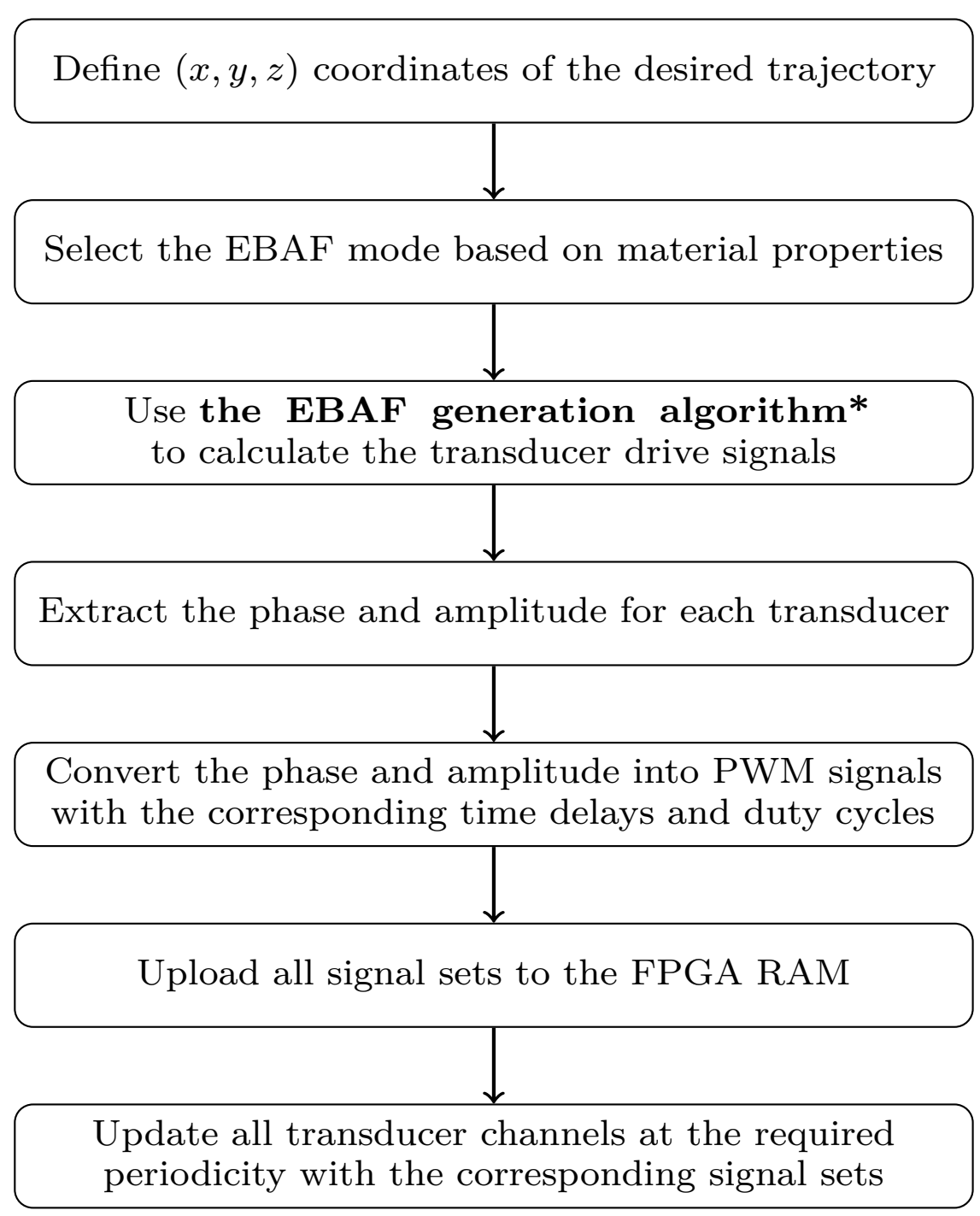

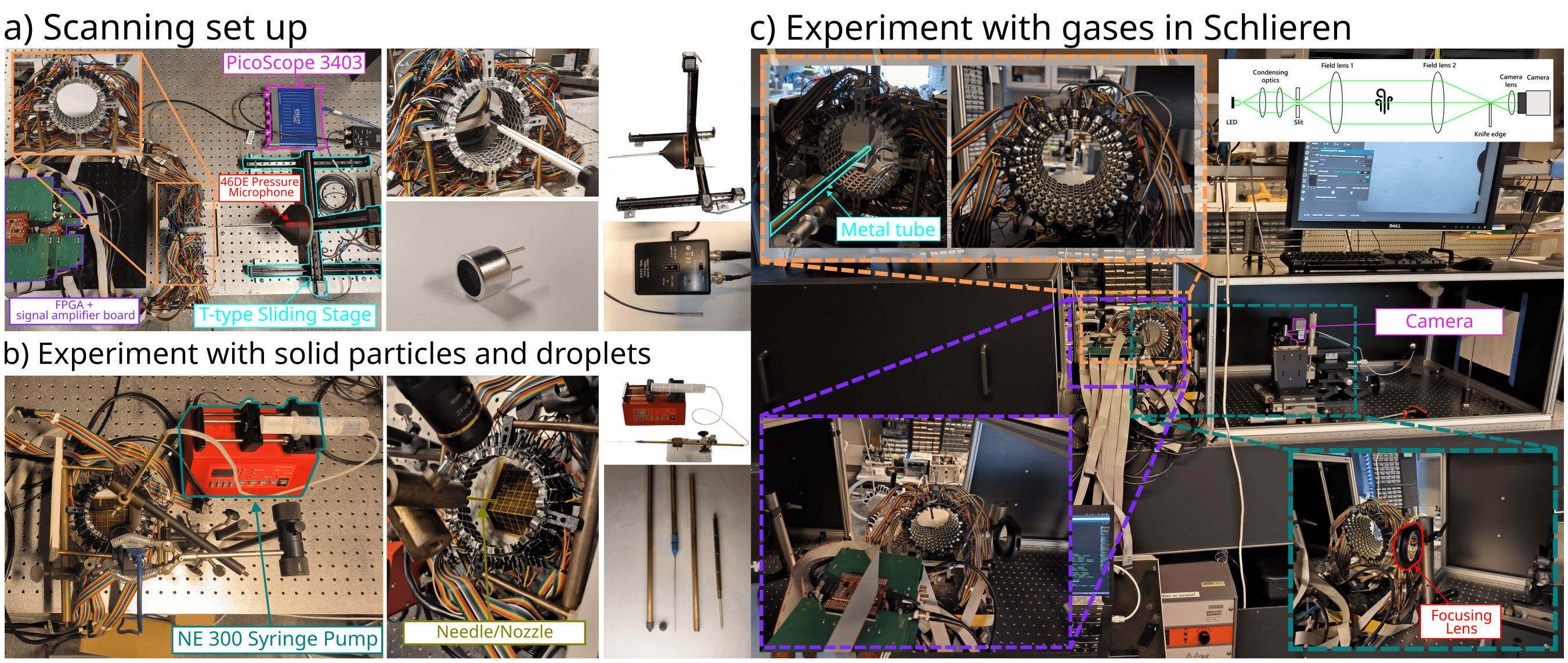

Fig. S1. Experimental setups. (a) Field scanning setup: A microphone mounted on a linear stage was moved point by point on a grid across the target region to measure the acoustic pressure at each point. (b) Setup for liquids and solids: Solid particles were introduced into the operation area through a nozzle, while liquids were pumped via a needle. The PAT was positioned vertically, and particle displacement was tracked using a camera and a scale paper placed at the bottom of the setup. (c) Setup for gases and aerosols: The PAT was oriented horizontally. For gas experiments, the observation area of the schlieren imaging system was positioned near the end of the array. For aerosol and ionized gas experiments, a camera for imaging was placed in front of the array.

Single emitter characterization

All transducers used for the PAT construction were required to have nearly identical amplitude and phase responses. However, the available airborne ultrasound transducers exhibited significant variations in these characteristics. To minimize differences in the emitted acoustic pressure signals of the set of transducers, a selection procedure was applied: the acoustic pressure of each transducer was measured at a distance of 10 cm from its surface when driven with CW square signal with $U_{pp} = 30\,\mathrm{V}$ peak-to-peak amplitude at 40 kHz. All measurements were performed using a single electronic channel used later for driving the PAT. Transducers with a phase deviation smaller than $\pi/4$ from the average phase and an amplitude within 75–100% of the maximum measured amplitude were selected for PAT construction.

To simulate the acoustic pressure field generated by the PAT, the amplitude and phase responses of all transducers in the array are required. In this work, for simplicity, all emitters were approximated as having identical amplitude and phase responses, taken from a representative transducer. The representative transducer was selected as the one whose amplitude response was closest to the average of the entire set. Its characteristics were measured at a distance of 10 cm by sweeping the frequency of CW square voltage signal of $U_{pp} = 30\,\mathrm{V}$ amplitude across 38–43kHz (Fig.S2d). The emitter efficiency was found to be 3.2 Pa/V at 40 kHz at 1 m distance, referenced to the peak-to-peak value of the applied square-wave signal.

Another defining characteristic of transducer emission is its directivity function. In this work, the directivity of all transducers was approximated by that of an idealized circular piston source, parameterized by its radius. To determine this, the acoustic pressure amplitude and phase of a representative transducer were measured over a transverse plane at 40 kHz (Fig.S2f) when driven by CW square voltage signal of 15 V peak-to-peak amplitude. In the measurements, the transducer was positioned with its surface normal to the measuring plane (Fig.S2h). To approximate the experimental field, the acoustic pressure distribution of an ideal circular piston source was simulated in a transverse plane located at the same distance as in the experiment, using the following equation:

$$P(x, y) = \frac{2J_1\left(k_0 a \sin\psi_k\right)}{k_0 a \sin\psi_k} \frac{\exp\left(-ik_0 R\right)}{R}, \qquad [1]$$

where a is the piston source radius, $R = \sqrt{x^2 + y^2 + h^2}$ is the distance from the center of the piston source to the observation point on the simulated plane, h is the axial distance between the transducer and the plane of the measurements, $\psi_k = \arctan(\sqrt{x^2 + y^2}/h)$ is the angle between the normal to the transducer and the line connecting the source center to the observation point.

The acoustic field was simulated on the same grid as that used for the experimental scanning of the transducer field. Both the experimental and simulated fields were normalized to their respective maximum values. The difference between the two fields was quantified by calculating the point-by-point differences of the absolute values of their complex amplitudes. The total sum of these differences was then minimized by varying the effective diameter of the piston source.

Let $\{(x_i, y_i)\}_{i=1}^{M}$ be the points on the scan grid and denote the amplitudes of the complex amplitudes of the experimental and simulated pressure field by $P_i^{(\mathrm{exp})}$ and $P_i^{(\mathrm{sim})}(a)$, respectively. To determine the effective piston radius, the following error functional was minimized:

$$E_2(a) = \sum_{i=1}^{M} \left(|P_i^{(\mathrm{exp})}| - |P_i^{(\mathrm{sim})}(a)| \right)^2. \qquad [2]$$

The minimization resulted in an effective piston source radius of a =4.6 mm.

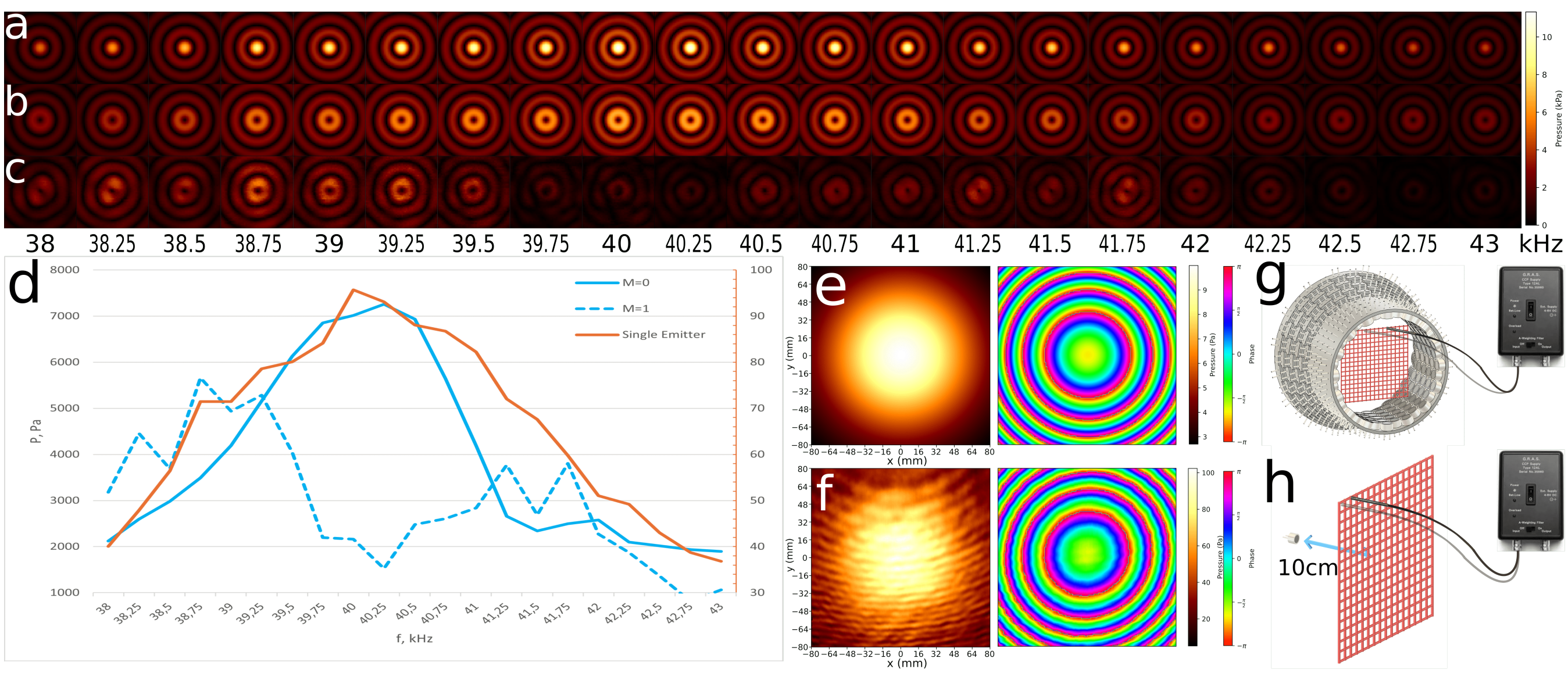

Fig. S2. (a,b) Simulated cross-sectional distributions of straight EBAFs in the center of cylindrical section of the PAT for the modes $m = 0$ and $m = 1$, respectively. (c) Corresponding experimental measurements of the mode $m = 1$. Both simulations and measurements were performed in the frequency range of 38–$43\,\mathrm{kHz}$ with a step of $0.25\,\mathrm{kHz}$. The experimental scans were carried out as shown in (g), over a square plane of size 25 mm with a step of 0.5 mm in the center of cylindrical segment of PAT. (d) The orange line shows the measured pressure amplitude at a distance of $10\,\mathrm{cm}$ in front of a single emitter driven by a $30\,\mathrm{V}$ square-wave signal. The blue solid and dotted lines show the maximum pressure amplitudes obtained from transverse scans for the $m = 0$ and $m = 1$ modes, respectively. (e,f) Normalized acoustic pressure amplitude and phase distributions at a plane located $10\,\mathrm{cm}$ in front of the emitter, obtained from simulations and measurements, respectively. The measurements were performed as shown in (h), over a square area of $160\,\mathrm{mm}$ side with a step size of $1\,\mathrm{mm}$.

Acoustic field scanning

Field scanning in a volume and along planes was performed using a microphone (46DE 1/8" CCP Pressure Microphone, GRAS, Denmark) connected to a digital oscilloscope (PicoScope 3403D, Pico Technology, United Kingdom). The microphone was mounted on a linear XYZ translation stage (T-type Sliding Stage, Toseastars, China) driven by a stepper motor (Nema 11 Stepper Motor, Toseastars, China).

Figure S3 presents a comparison between the theoretical (computed via Eq. (8)) and experimentally scanned field distributions of various EBAF modes. The scanning was done in the middle of PAT as depicted in Fig.S2g. The results demonstrate strong agreement between theory and experiment.

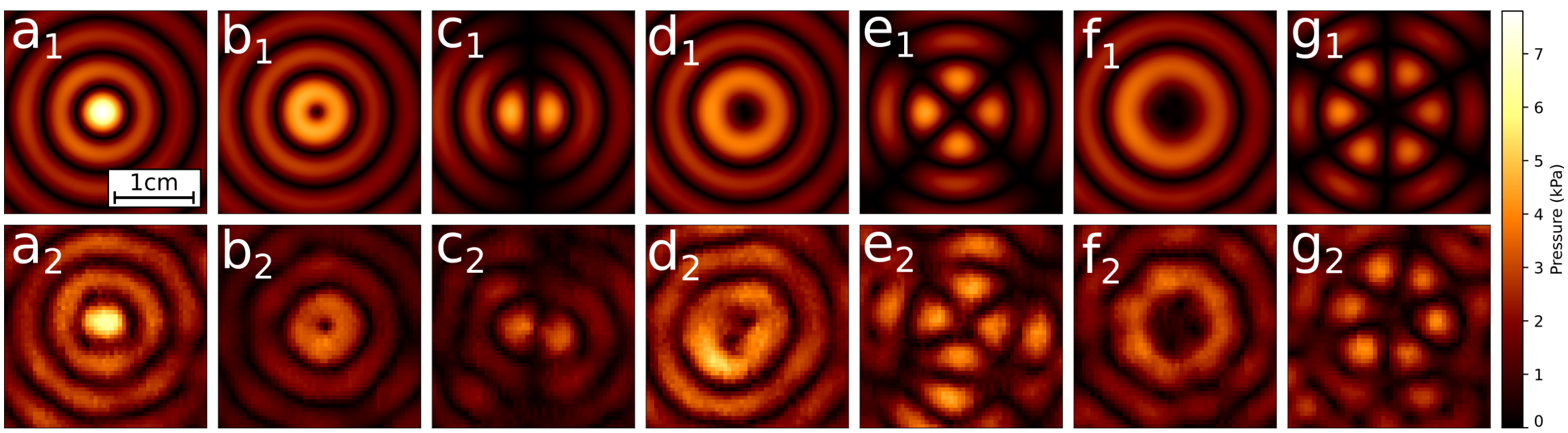

Fig. S3. Top row: simulated fields; bottom row: corresponding experimental measurements. Modes shown from left to right: $m = 0$, $m = 1$, $m = \pm 1$, $m = 2$, $m = \pm 2$, $m = 3$, and $m = \pm 3$. Measurements were performed over a $25\,\mathrm{mm} \times 25\,\mathrm{mm}$ square plane with a spatial step of $0.5\,\mathrm{mm}$, positioned at the center of the cylindrical section of the PAT. Simulations were carried out in the same plane for direct comparison.

Method for generation of EBAFs shaped along 3D paths

The workflow of **the EBAF generation algorithm*** is shown below:

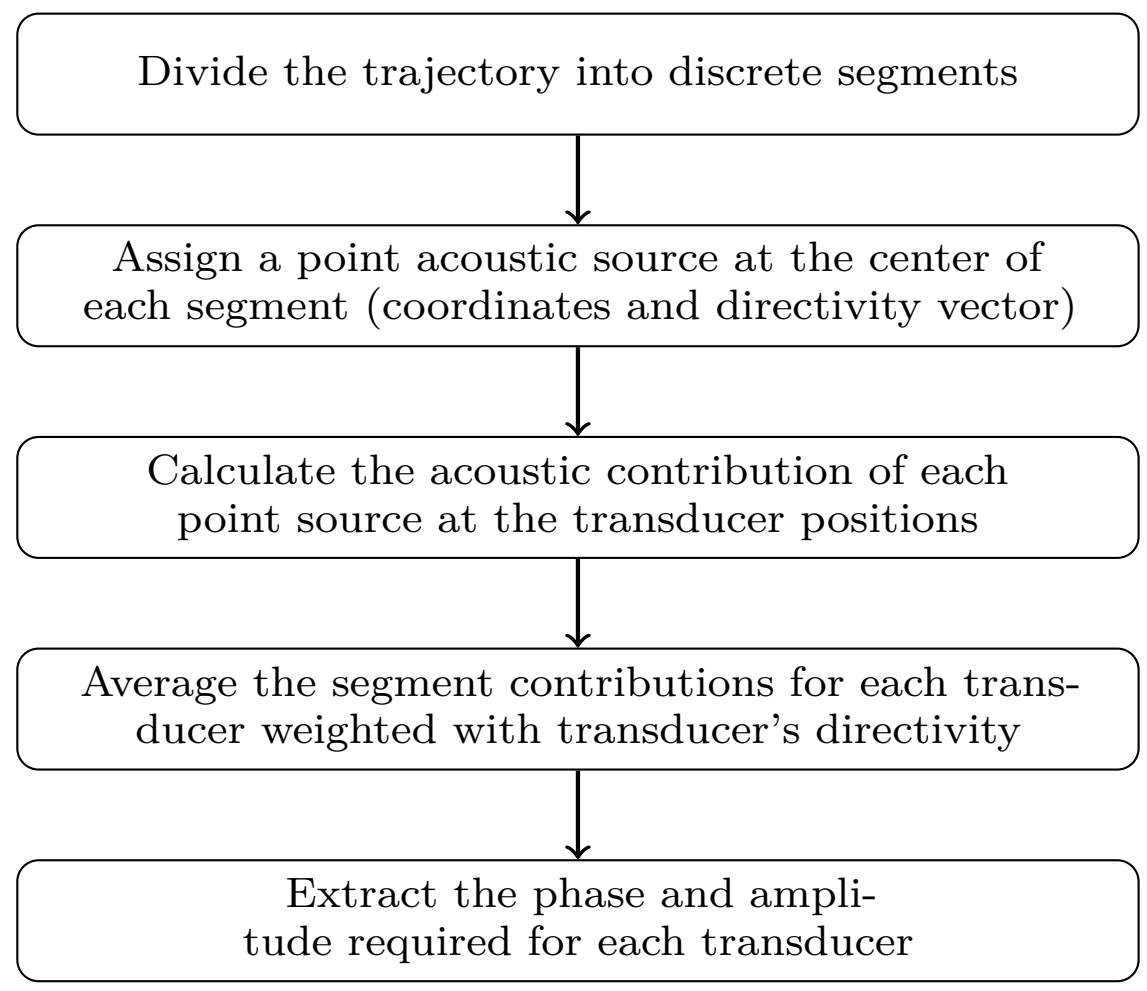

Elementary point source radiation. The acoustic field generated by a finite-sized body at the origin, radiating a monochromatic wave, can be expressed as a series expansion in spherical multipole components (1):

$$P(r,\theta,\varphi) = \sum_{n=0}^{\infty}\sum_{m=-n}^{n} C_{mn} h_n^{(1)}(k_0 r)\, Y_n^m(\theta,\varphi),$$
$$Y_n^m(\theta,\varphi) = \sqrt{\frac{(2n+1)(n-m)!}{4\pi(n+m)!}} P_n^m(\cos\theta)\exp(im\varphi), \quad [3]$$

where r is the radial distance to the observation point, which is assumed to be outside the smallest sphere enclosing the source. The angles θ and φ are the polar and azimuthal coordinates, respectively. The functions $P_n^m(\cos\theta)$ are the associated Legendre polynomials, C_{mn} are the multipole expansion coefficients, $k_0 = \omega/c_0$ is the acoustic wavenumber, ω is the angular frequency of the radiated signal and c_0 is the speed of sound. $h_n^{(1)}(\cdot)$ is the spherical Hankel function of the first kind.

Each individual term in Eq. (3) corresponds to a multipole component expressed as

$$P_n^m(r,\theta,\varphi) = C_{mn} h_n^{(1)}(k_0 r) Y_n^m(\theta,\varphi), \quad [4]$$

with the lowest-order cases representing the fundamental acoustic sources:

1) Monopole - the zeroth-order term ($n = m = 0$) radiates spherically in all directions (Fig.S4 e).

2) Dipole radiation along the z-axis (corresponding to $\theta = 0$) arises from the $n = 1$, $m = 0$ multipole, p_1^0. Dipoles oriented along the x- and y-axes (corresponding to the $\theta = \pi/2$, $\varphi = 0$ and $\theta = \pi/2$, $\varphi = \pi/2$) are obtained from the real and imaginary parts of the $n = 1$, $m = 1$ multipole, i.e. $\mathrm{Re}(p_1^1)$ and $\mathrm{Im}(p_1^1)$, respectively (Fig.S4 e).

3) Point vortex of mode K corresponds to the multipole term with $m = n = K$, where both the real and imaginary parts of p_K^K contribute to the rotational structure of the field. Radiation patterns for point vortices with modes $m = 0, 1, \pm1, 2, \pm2$ are shown in Fig.S4 e.

Virtual EBAF source. To generate an EBAF, an appropriate boundary condition should be specified on the surface Σ enclosing the source region. This boundary condition can be derived from the radiation of a virtual acoustic source.

A virtual source corresponding to an EBAF of mode m, following a prescribed curved trajectory, can be represented as a superposition of point sources distributed along that path as shown in Fig.S4 a. Each point source radiates according to the multipole term p_m^m from Eq. (4). The trajectory of total length L is divided into N equal-length segments, each much shorter than the wavelength ($L/N \ll \lambda$). A point multipole is positioned at the center of each segment. The total acoustic field on Σ is then obtained by summing the contributions from all point multipoles:

$$P(\mathbf{r}) = C\sum_{n=1}^{N} h_m^{(1)}(k_0\|\mathbf{r}-\mathbf{r}_n\|) Y_m^m(\theta_n,\varphi_n), \quad [5]$$

where $\mathbf{r} = (r,\theta,\varphi)$ is the coordinates of the calculation point, C is a scaling coefficient, $\mathbf{r_n}$ denotes the position of the n-th multipole along the prescribed trajectory.

The polar angle θ_n is defined as the angle between the vectors $\mathbf{a_n} = (\mathbf{r_{n+1}} - \mathbf{r_n})/\|\mathbf{r_{n+1}} - \mathbf{r_n}\|$ and $R_n = (\mathbf{r} - \mathbf{r_n})/\|\mathbf{r} - \mathbf{r_n}\|$:

$$\theta_n = \arccos(\mathbf{a_n} \cdot \mathbf{R_n}) \qquad [6]$$

where $\mathbf{a_n}$ is the unit tangent to the EBAF trajectory at the n-th point, and $\mathbf{R}_n$ is the unit vector pointing from the n-th point source position $\mathbf{r}_n$ to the field calculation point $\mathbf{r} = (r, \theta, \varphi)$ on the surface Σ.

The azimuthal angle φ_n is defined as

$$\varphi_n = \arccos\left(\mathbf{R_{n\perp}} \cdot \mathbf{e_{x\perp}}/(\|\mathbf{R_{n\perp}}\|\|\mathbf{e_{x\perp}}\|)\right), \qquad [7]$$

where $\mathbf{R_{n\perp}} = \mathbf{R_n} - (\mathbf{a_n} \cdot \mathbf{R_n})\mathbf{a_n}$ and $\mathbf{e_{x\perp}} = \mathbf{e_x} - (\mathbf{a_n} \cdot \mathbf{e_x})\mathbf{a_n}$ are the projections of $\mathbf{R_n}$ and the x-axis unit vector $\mathbf{e}_x$ onto the plane perpendicular to the vector $\mathbf{a}_n$.

As an illustrative example, Fig. S4 presents the field distributions on the unfolded boundary surface of the PAT corresponding to the generation of EBAFs along a trajectory composed of two straight segments. The results are shown for both amplitude and phase of the complex acoustic pressure field. Specifically, Fig. S4b1,c1 correspond to the mode $m = 0$, while Fig. S4f1,g1 correspond to the mode $m = 1$.

Simulation of the acoustic pressure field generated by the PAT. For numerical field reconstruction, the conjugated complex pressure amplitude is prescribed on the boundary surface enclosing the source, with the boundary chosen to be as large as possible, following the acoustic holography approach (2). For physical field reconstruction, this boundary can be discretized into a set of point sources with spacing smaller than half a wavelength, each radiating with the corresponding amplitude and phase. In the present work, the PAT elements themselves are these point sources surrounding the EBAF. According to Eq. (5), the acoustic pressure is calculated at the positions $\mathbf{r}_k$ corresponding to the centers of the k-th transducers. An example of the resulting pressure amplitude and phase distributions of the transducers is shown in Fig. S4b2,c2,f2,g2.

Having obtained the complex pressure amplitude P_k on all K transducers, the corresponding particles velocity were approximated assuming plane progressive waves, giving $V_k = P_k/(\rho c_0)$, where ρ and c_0 are the density and sound speed of air, respectively. The acoustic field can be reconstructed using the piston model of the transducer radiation (3). The pressure at a point $\boldsymbol{r}$ is expressed as

$$\begin{aligned} P(\boldsymbol{r}) &= -\omega^2 \rho a^2 \sum_{k=1}^{K} V_k^* D\left(\psi_k\right) \frac{\exp\left(-ik_0\|\boldsymbol{r} - \boldsymbol{r}_k\|\right)}{\|\boldsymbol{r} - \boldsymbol{r}_k\|}, \\ D\left(\psi_k\right) &= \frac{2J_1\left(k_0 a \sin\psi_k\right)}{k_0 a \sin\psi_k}, \end{aligned} \qquad [8]$$

where $\psi_k = \arccos(\boldsymbol{n}_k \cdot \boldsymbol{R}_k)$ is the angle between the unit vector $\boldsymbol{R}_k$ and the normal to the k-th transducer $\boldsymbol{n}_k$. The operator $(.)^*$ denotes complex conjugation. The function $D(\cdot)$ represents the piston source directivity, with $J_1(\cdot)$ being the Bessel function of the first kind, a the transducer radius, and $k_0 = \omega/c_0$ the acoustic wavenumber.

Equivalently, the coefficient $-\omega^2 \rho a^2 V_k^*$ in Eq. (8) can be replaced by $P_0 \times e^{i\varphi}$, where P_0 is the pressure amplitude of a transducer at a distance of 1 m, and φ is the phase of the complex pressure amplitude P_k^*.

In the calibration procedure, P_0 was measured with the transducers driven by a continuous-wave (CW) square signal with 30 V peak-to-peak amplitude (the maximum voltage used in this work) and then referenced to the applied voltage. For an arbitrary drive peak-to-peak voltage amplitude U_{pp}, the effective P_0 scales linearly as $P_0 \times U_{pp}$, since both the vibrational velocity and the generated pressure amplitude are proportional to the applied voltage over a wide range. This proportionality provides a straightforward means to control the pressure value of the EBAF by uniformly adjusting the transducer drive voltages.

The pressure distributions calculated with Eq. (8), using the PAT driving signals derived for curved EBAFs of modes $m = 0$ and $m = 1$ according to Eq. (5), are shown in Fig. S4d,h, respectively.

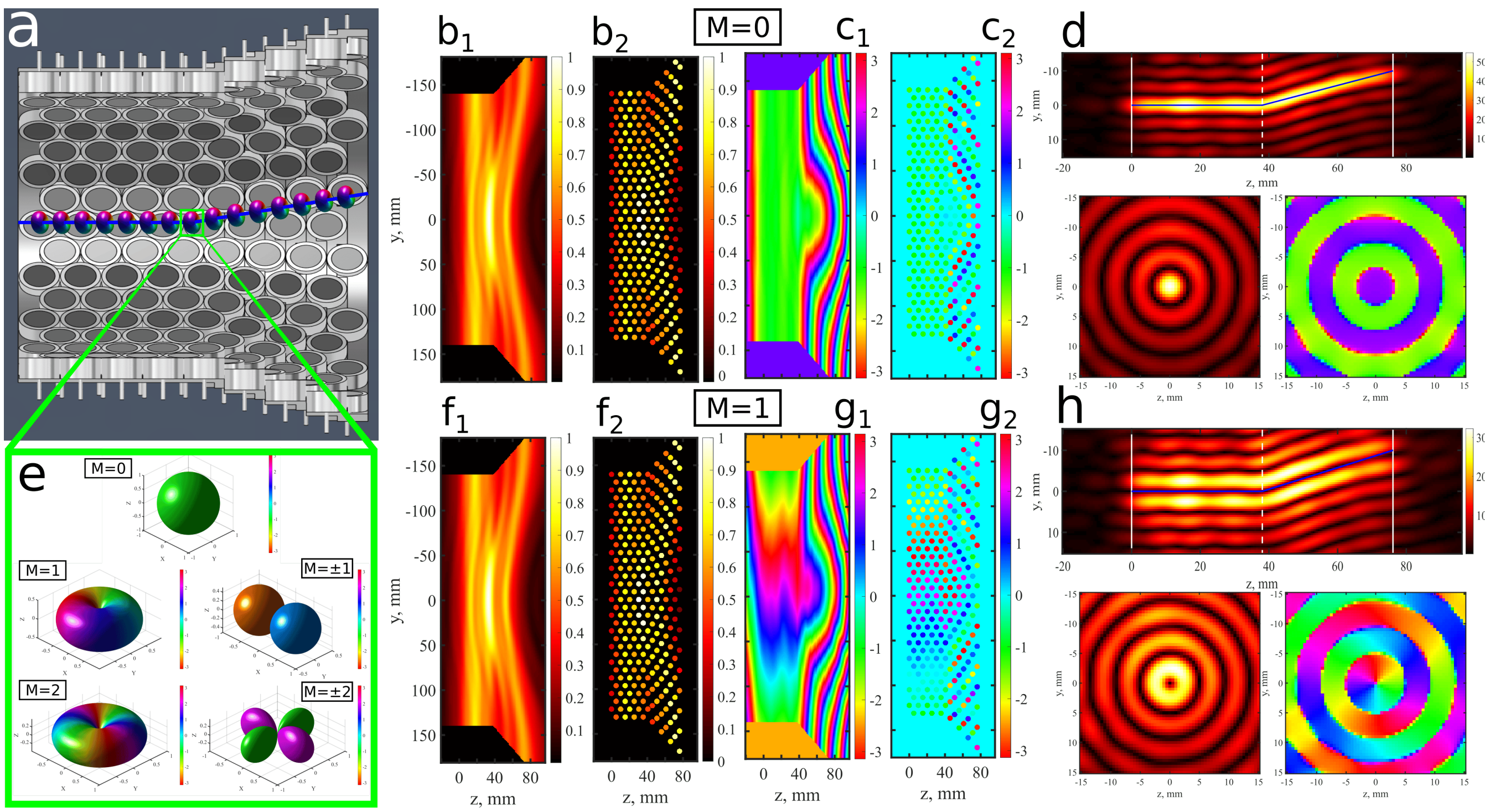

Fig. S4. Illustration of the EBAF generation algorithm. (a) PAT with elementary point sources distributed along the desired path. (e) Elementary point sources of the modes $m = 0, 1, 2, \pm1, \pm2$. (b1,c1) Normalized acoustic pressure amplitude and phase distributions on the surface of the PAT obtained with the EBAF generation algorithm for mode $m = 0$. (f1,g1) Same as (b1,c1) but for mode $m = 1$. (b2,c2,f2,g2) Corresponding pressure amplitudes and phases of the PAT emitter drive signals obtained with the EBAFs generation algorithm. (d,h) Acoustic pressure amplitude and phase calculated using the field generation algorithm for modes $m = 0$ and $m = 1$, respectively.

Pseudo-code for EBAFs generation algorithm. The pseudocode implementing the above-described method for calculating the drive signals of the transducers is shown in Algorithm 1. Note the use of the complex argument in line 23 to generate the proper phases.

One can evaluate special cases of Y_{mn}, for example, when $m = n = 1$, by using Eq. 9 (1) and not calling a general Y_{mn} function, for example, `lpmn()` in the scipy library to get the sequence of associated Legendre functions of the first kind. This significantly decreases the algorithm's computation time.

$$Y_{11}(b, \phi) = -\sqrt{\frac{3}{8\pi}}\sqrt{1-b^2}\; e^{j\phi} \qquad [9]$$

Algorithm 1 EBAF forward calculation

1: Check that z-components of path points are in decreasing order

2: ▷ Calculate a_n unit vectors tangent to path using path points stored in p_{path}
3: Check that # of path points is > 3
4: Make 2D array a_n of dimensions # of path points by 3 to store normal vectors

5: $\Delta p = p_{path}[0,\ :] - p_{path}[1,\ :]$
6: $a_n[0,\ :] = \Delta p/|\Delta p|$

7: **for** $n = 1$ to # of path points - 2 **do**
8: $\quad \Delta p = p_{path}[n-1,\ :] - p_{path}[n+1,\ :]$
9: $\quad a_n[n,\ :] = \Delta p/|\Delta p|$

10: $\Delta p = p_{path}[-2,\ :] - p_{path}[-1,\ :]$
11: $a_n[\#paths - 1,\ :] = \Delta p/|\Delta p|$

12: Make 1D array of zeros s with length equal to # of transducers
13: $v_{n,\phi} = [0, 1, 0]$ ▷ This unit vector is constant if the path is generated on the xz-plane

14: **for** $k = 0$ to # of transducers - 1 **do**
15: $\quad$ **for** $n = 0$ to # of path points - 1 **do**
16: $\quad\quad v_{pt} = p_{transducer}[k,\ :] - p_{path}[n,\ :]$
17: $\quad\quad d_{pt} = |v_{pt}|$

18: $\quad\quad$ ▷ θ calculation
19: $\quad\quad a = v_{pt} \cdot a_n[n,\ :]$
20: $\quad\quad b = a/d_{pt}$ ▷ Cosine of θ

21: $\quad\quad$ ▷ Φ calculation (using this method to clamp values between $-\pi$ and π)
22: $\quad\quad c_n = a_n[n,\ :] \times v_{n,\phi}$
23: $\quad\quad \phi = \angle\{[-v_{pt} \cdot (v_{n,\phi} + jc_n)]/d_{pt}\}$
24: $\quad\quad p_k = H1_n(m, k_0 d_{pt}) Y_{mn}(b, \phi)$

25: $\quad\quad s[k,\ :] = s[k,\ :] + p_k$

26: $s = s/max(|s|)$

Pseudo-code of the field generation algorithm. The pseudocode implementing the method for calculating the acoustic field distribution radiated by the PAT is shown in Algorithm 2. The total pressure at a set of sampled points is found by adding the pressure due to each transducer, after EBAF signal for each transducer is applied.

Algorithm 2 Sound field simulation

Make 1D array of zeros p with length equal to # of transducers
to store complex pressure values at each sampled point

for $n = 0$ to # of sampled points - 1 **do**
$\quad$ **for** $k = 0$ to # of transducers - 1 **do**

$\quad\quad$ ▷ Add pressure contribution for each transducer by using complex signal
$\quad\quad$ found from Algorithm 1 and evaluating Eq. 8
$\quad\quad p[n,\ :] = p[n,\ :] + p(s^*[k], \psi_k[k], r_k[k], r[n])$

Effect of amplitude modulation for EBAFs generation

The EBAFs generation algorithm computes the complex pressure amplitudes for driving the transducers, including both phase and amplitude components. In most studies, only the phase component of the excitation signal is used with a uniform

amplitude applied across all transducers. This is usually sufficient for generating localized point traps. In contrast, the acoustic field targeted in the present work is considerably more complex, extending along a prescribed path rather than being confined to a single point. In such cases, the amplitude distribution across the transducers plays a crucial role in ensuring the uniformity of the generated field. Therefore, both phase and amplitude information should be incorporated into the drive signals.

As shown in Fig.S5, including the amplitude component in the drive signals significantly improves the uniformity of the acoustic pressure along the EBAF trajectory. In contrast, neglecting amplitude modulation leads to non-uniform pressure distributions, resulting in low-pressure regions along the EBAF. These regions act as weak points in the field and may cause 'leakage' of the manipulated matter during guiding.

Amplitude control over the transducers reduces the generated pressure (Fig. S5) compared to applying the same voltage amplitude to all transducers, since the average voltage in the amplitude control case is lower. In practice, these two alternatives lead to distinct physical effects:

1. Using no amplitude modulation leads to maximized pressure and ARF, enabling stronger guiding capabilities but resulting in less predictable particle motion due to field nonuniformity. 2. Using amplitude modulation reduces the average pressure and ARF, leading to weaker guiding but more predictable and confined particle motion.

In this work, we used the first approach, operating without amplitude modulation. Nevertheless, the efficiency of material guidance with EBAFs could be further enhanced by implementing amplitude control, provided that higher drive voltages are supplied through more advanced electronics to compensate for the reduced pressure.

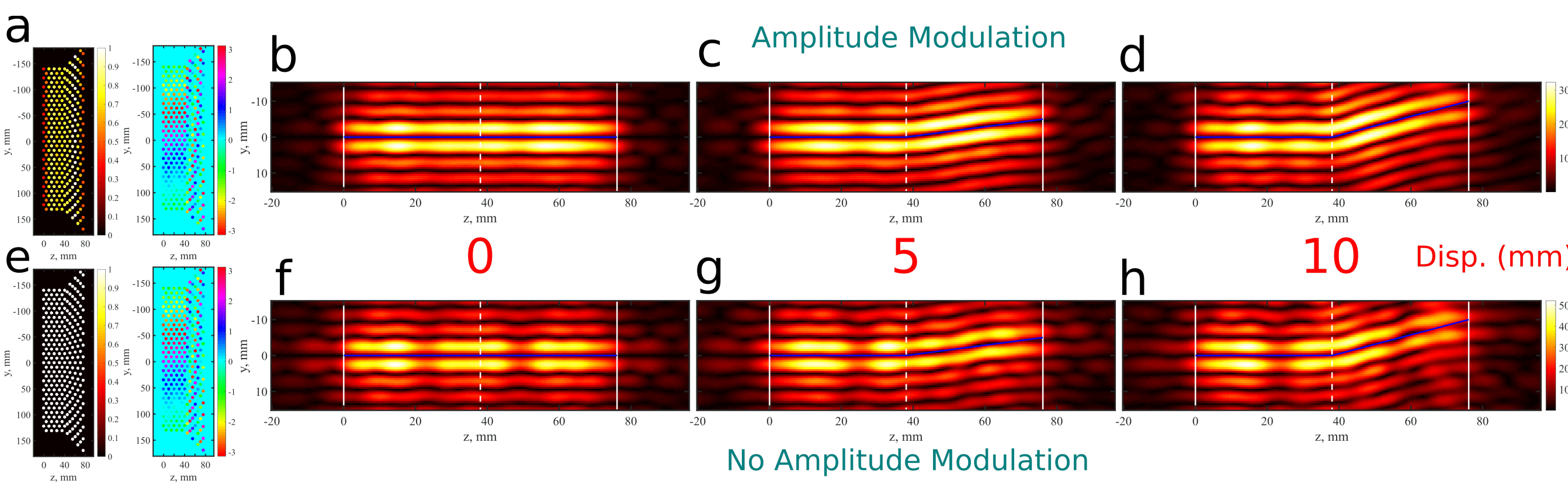

Fig. S5. Effect of amplitude control of the transducer signals. (a,e) Amplitude and phase distributions of the emitter driving signals for a straight EBAF of mode $m = 1$, with (a) and without (e) amplitude control. In the case without amplitude control, all emitters are driven with equal amplitudes. (b–d) Acoustic fields calculated using the field generation algorithm with amplitude control for EBAFs with trajectory displacements of 0, 5, and $20\,\mathrm{mm}$ at the PAT end, respectively. (f–h) Same as (b–d), but without amplitude control applied to the emitters.

Rationale for the geometry of the array

The cylindrical-conical geometry was developed based on observations obtained during array prototyping. The initial design consisted of cylindrical sections, which provided a modular architecture in which the array length could be scaled by stacking individual segments. This configuration reliably generated axis-aligned EBAFs. However, when EBAFs were intentionally curved a significant reduction in the control of the flow direction of the guided material was observed.

This behavior was attributed to multiple reflections within the cylindrical cavity, which can interfere with the primary acoustic field and thereby reduce control over the intended field formation. A conical section was therefore introduced to provide an expanding geometry in which reflected acoustic waves can propagate away from the active guidance region, reducing the influence of multiple reflections on the synthesized field.

The effect of the geometry was characterized by comparing the acoustic fields generated by (1) a purely cylindrical array, composed of two stacked cylindrical segments, and (2) the cylindrical-conical array, composed of one cylindrical and one conical segment. In both configurations, tilted EBAFs corresponding to displacements of 0, 5, 7.5, and 10 mm were synthesized along the second segment, which was cylindrical or conical, respectively. The conical geometry produced a more uniform acoustic field along the curved guidance path, with fewer regions of strongly reduced acoustic pressure (Fig. S6). This improvement was particularly pronounced in the central region of the EBAF, where the acoustic trajectory begins to tilt. Moreover, the experimentally measured field distribution obtained with the conical geometry (Fig. S6b2-b5) showed closer agreement with the simulated acoustic field (Fig. S6a2-a5). The cylindrical-conical configuration consequently provided more stable and reliable material guidance, particularly during EBAF bending.

For the gas and aerosol experiments (Fig. 4) and the horizontal water-steering experiments (Fig. 1g-i), the complete cylindrical-conical array was used. In these configurations, the initial cylindrical section provided axis-aligned material delivery toward the trajectory-steering region of the EFAFs. All other experiments were performed using only the conical section.

The geometric parameters of the cylindrical-conical configuration were determined primarily by practical design considerations. The array dimensions and transducer arrangement were chosen to maximize the generated acoustic pressure while maintaining a practical material-guidance length. The total number of transducers was constrained by the available channels of the driving electronics. The diameter of the cylindrical section and the minimum aperture of the conical section were set to 89 mm which is the smallest practical dimensions compatible with the physical transducer size and the used circumferential arrangement of 16 transducers. This configuration maximized the transducer packing density and, consequently, the achievable acoustic pressure amplitude, while maintaining the desired guidance length and overall array geometry.

Along the array axis, the transducers were arranged with an inter-element pitch of 5.08 mm, that was close to half of the acoustic wavelength ($\lambda/2 = 4.3$ mm). In phased array design, maintaining inter-element spacing below or near ($\lambda/2 = 4.3$) is crucial for suppressing spatial grating lobes and ensuring precise control over the synthesized acoustic field (4). This criterion was therefore used to define the target axial spacing between adjacent transducers.

The achievable pitch was also constrained by the physical dimensions of the transducers, which had a diameter of 9.8 mm. To maximize the transducer packing density, adjacent rings were circumferentially shifted so that neighboring transducers could be positioned closer than the transducer diameter. This allowed the minimum axial spacing to be reduced to half of the transducer diameter equal to 4.9 mm. A slightly larger spacing of 5.08 mm was ultimately selected to provide sufficient structural integrity while remaining close to the ($\lambda/2 = 4.3$) design criterion.

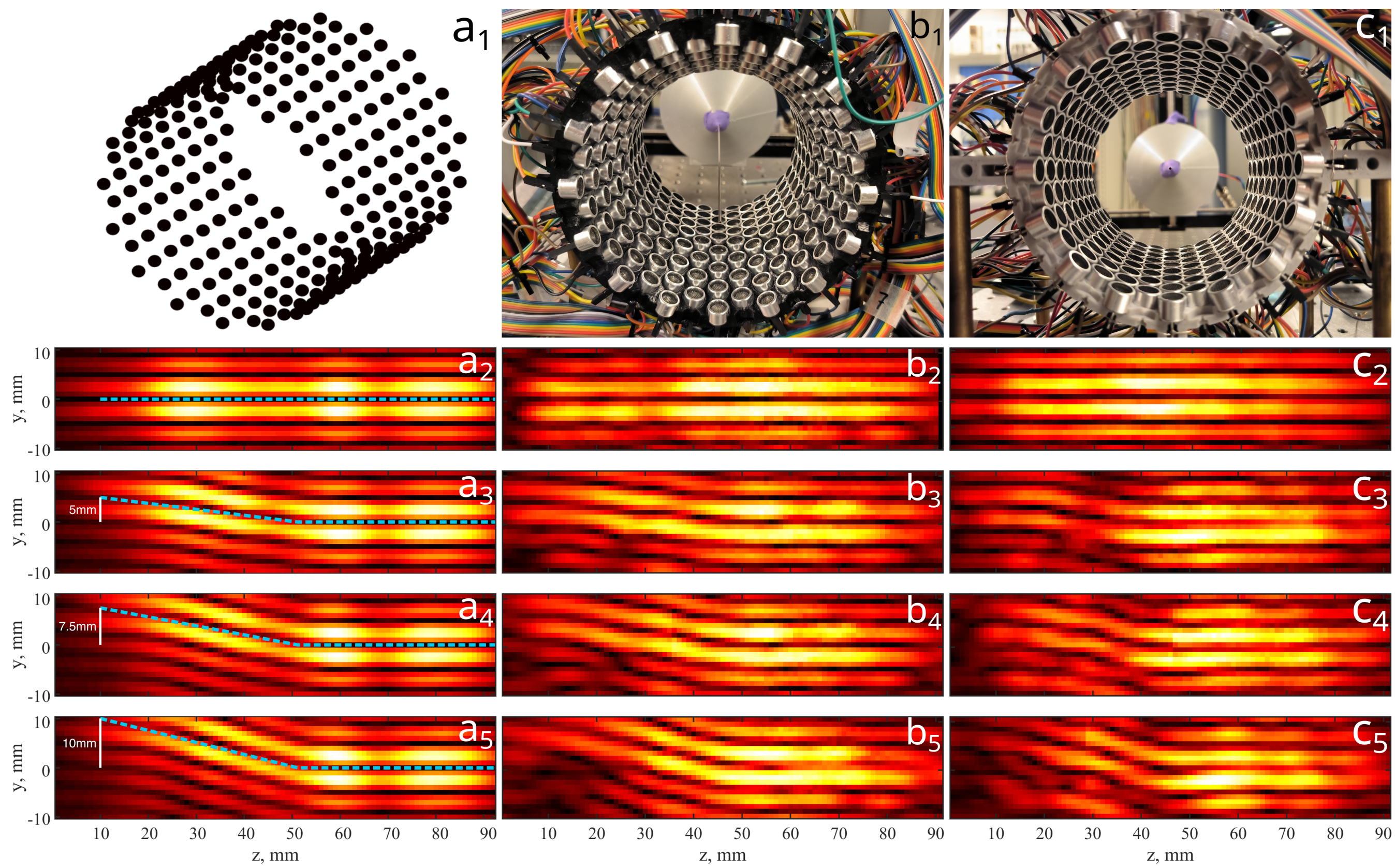

Fig. S6. Comparison of the acoustic fields generated by (b1) the cylindrical-conical and (c1) cylindrical-cylindrical array geometries. (a2-a5) Simulated acoustic fields for (a1) the cylindrical-cylindrical geometry at displacements of 0, 5, 7.5, and 10 mm, respectively. (b2-b5) Experimentally scanned acoustic fields generated by the cylindrical-conical geometry at displacements of 0, 5, 7.5, and 10 mm, respectively. (c2-c5) Experimentally scanned acoustic fields generated by the cylindrical-cylindrical geometry at the corresponding displacements of 0, 5, 7.5, and 10 mm, respectively.

Effect of acoustic streaming on guiding gasesous media

In the experiments, pronounced instabilities were observed for powder particles, aerosols, and gases when guided with the modes $m = 1, 2, \ldots$, whereas large particles exhibited stable guidance. This effect has received little attention in previous studies, and its underlying cause remains unclear. In this work, we attribute the observed instabilities to stationary flows induced by acoustic streaming. To clarify the role of streaming in the guiding performance, acoustic streaming was numerically simulated using the finite element method (FEM) for various field configurations produced by the PAT.

Simulation of acoustic streaming. The simulations were performed in three dimensions, however, due to high computational cost, the model was simplified to include only the cylindrical section of the PAT responsible for generating a straight part of the EBAF. Straight EBAFs of modes $m = 0, 1, \pm 1$ were synthesized, with the acoustic pressure magnitude matched to the experimental value (Fig.2) with a maximum pressure amplitude of 5.3 kPa.

The model was implemented in COMSOL Multiphysics 6.2 (COMSOL AB, Stockholm, Sweden). The acoustic field was obtained by solving the Helmholtz equation, with the cylindrical boundary excited using a normal displacement condition. The ends of the cylindrical pipe section were modeled as perfect acoustic absorbers. The phase of the radiating surface was prescribed using the EBAFs generation algorithm, and the amplitude was scaled to get the pressure value equal to the experimental one 5.3 kPa. The resulting acoustic field served as a source term for the acoustic streaming simulation, which was modeled as laminar flow with a no-slip boundary condition on the cylindrical surface and an open boundary on the ends of the cylindrical surface. Thermoviscous dissipation was included as the primary loss mechanism, where attenuation arises from both thermal and viscous effects in the bulk fluid. The thermoviscous attenuation coefficient was defined using the Stokes–Kirchhoff relation. Coupling between the acoustic field and fluid flow was introduced through COMSOL's acoustic streaming module. Thermal effects, material property gradients, and their contributions to streaming were not included.

The simulations revealed that the $m = 1$ mode generated acoustic streaming with magnitudes nearly two orders of magnitude higher than those of $m = 0$ and $m = \pm 1$, with maximum velocities approaching $200\,\mathrm{mm\,s^{-1}}$. In addition, the $m = 1$ mode produced a pronounced rotational streaming pattern, consistent with the experimentally observed vortex-like flow (Fig.S7c2). In contrast, the $m = 0$ and $m = \pm 1$ modes produced only weak streaming (Fig.S7c1,c3), with maximum velocities approximately 12 and $30\,\mathrm{mm\,s^{-1}}$ and without significant rotational components capable of disrupting the guided medium.

Gor'kov potential of the EBAFs. The corresponding Gor'kov potentials of the modeled fields are shown in Fig. S7b1,b2,b3. The air supplied by the ultrasonic humidifier is cooled due to the adiabatic evaporation of droplets. As reported in (5), the aerosol stream locally cools, and the cooling can reach 2–3°C depending on operating conditions (airflow rate, humidity, and mist loading). For the potential calculation, the aerosol air temperature was set 2 °C lower than the ambient air under standard conditions. The ambient air was assumed to have density $\rho = 1.2\ \mathrm{kg\,m^{-3}}$ and speed of sound $c = 343\ \mathrm{m\,s^{-1}}$, whereas the cooled air has properties $\rho = 1.21\ \mathrm{kg\,m^{-3}}$, and $c = 342.2\ \mathrm{m\,s^{-1}}$. The Gor'kov potential of this cooled air in the surrounding ambient air was calculated following the approach described in (6).

The Gor'kov potential provides an effective representation of the acoustic field for analyzing gas transport, since gases tend to migrate toward regions of low potential due to ARF. This allows identification of the flow pathways of the cooled air within the field. For instance, in the mode $m = 0$, the gas is directed into a circular region surrounding the central acoustic pressure maximum directed along the path of EBAF, while in the modes $m = 1$ and $m = \pm 1$, the gas is directed along the axis of EBAF.

Experimental observation of the acoustic streaming of different modes. The guiding of aerosols was investigated in EBAFs of modes $m = 0, 1, \pm 1$ along a straight field aligned with the PAT axis (Fig.S7a1,a2,a3). The release pipe was aligned with the axis of the PAT, producing a laminar aerosol stream directed along the PAT axis (Fig.S7e). The observed aerosol behavior is shown in Fig.S7d1,d2,d3. Among the tested modes, $m = 0$ consistently guided the aerosol along the entire trajectory (Fig.S7d1), whereas $m = 1$ disrupted the flow and induced rotational motion (Fig.S7d2). The mode $m = \pm 1$ achieved only partial guiding, causing moderate flow disturbance, but less intense than that observed.

The results of the streaming simulations explain why the modes $m = 1, \pm 1$ failed to guide the gaseous medium. The streaming generated by $m = 1$ was strong with a significant rotational component which disrupts the laminar flow of gas and aerosol (Fig.S7c2). The mode $m = \pm 1$ appears suitable for guiding aerosols and gases, as it features a localized low-potential region along the axis (Fig.S7b3) and generates streaming velocities significantly lower than those of mode $m = 1$ (Fig.S7c3). However, in the simulations, the aerosol stream was directed toward the axis in a non-axisymmetric manner, with the flow compressing the aerosol from the sides (Fig.S7c3). This effect was sufficient to disturb the overall flow stability. Overall, the guiding efficiency of mode $m = \pm 1$ was lower than that of mode $m = 0$ but higher than that of mode $m = 1$.

Comparison of effects of acoustic streaming and ARF on gas guidance. To distinguish the contributions of acoustic radiation force and acoustic streaming two controlled conditions were studied using heavy gas and air flows. In the first condition, the acoustic contrast between the gas and the surrounding air was maximized, thereby enhancing the expected contribution of ARF. In the second condition, the acoustic contrast was minimized, reducing the expected contribution of ARF and allowing for the evaluation of the effects of acoustic streaming in ambient air and flow entrainment. High-contrast gas trans-1,3,3,3-tetrafluoropropene (E-isomer, HFO-1234ze, mass density $\rho = 4.7\mathrm{kg\,m^{-3}}$) and a control stream of room-temperature air ($\rho = 1.2\mathrm{kg\,m^{-3}}$) were used.

Both gases were introduced into the acoustic field under identical injection conditions (the flow rate was approximately equal 1 L/min) through a 5 mm internal diameter nozzle. The trajectory of the m=0 mode acoustic beam was dynamically

steered across a lateral displacement range from -10 mm to +10 mm in 1 mm spatial steps updated every 0.2 s similar to the experiment with N_2O gas (Movie S4).

Because conventional shadowgraphy cannot track room-temperature air streams due to the absence of optical refractive index contrast, we implemented a laser light-sheet visualization setup inside a fog-filled enclosure. The light-sheet illuminated the plane across which the acoustic beam was dynamically displaced.

As demonstrated in Movie S6, the HFO-1234ze plume is dynamically steered along the acoustic trajectory. In contrast, the room-temperature air stream exhibits no lateral deflection. For room-temperature air, the experiment for air was repeated across a broad range of flow rates (0.1 - 5 L/min), and no field-induced air streaming was observed.

The lack of comparable data for airflow at room temperature suggests that acoustic streaming alone cannot explain the observed behavior. Thus, for the $m = 0$ mode, the dominant mechanism is the acoustic radiation force associated with the acoustic contrast at the gas-air interface, while acoustic streaming and carrier flow entrainment make only minor contributions.

Experiment on guidance of different gases. To investigate the influence of gas properties on acoustic guidance, additional experiments were performed using gases with different densities under the same acoustic field conditions and comparable flow rates. The tested gases included denser than air gases (N_2O, CO_2, and cold air at 1°C) and lighter than air gases (He and heated air at 200°C). For all experiments, the same EBAF mode $m = 0$ and trajectory were used. The trajectory was dynamically steered by periodically applying transverse displacements, following the same procedure as in the experiment shown in Fig. 4. The denser than air gases exhibited stable and precise guidance. The injected streams remained confined to the EBAF and followed the dynamically changing trajectory. In contrast, He and heated air at 200°C showed no observable guidance by the acoustic field and dispersed over the surrounding area. This unguided behavior of He and heated air persisted over the investigated flow rate range of 0.1-5 L/min. Movie S6 illustrates the flow behavior in EBAF of heated air 200°C, and Movie S7 shows the corresponding dynamics of N_2O, CO_2, cold air at 1°C, and He.

FEM simulation of gas dynamics

To further investigate the interaction between the gas and the acoustic field, we developed a two-dimensional multiphysics finite-element model in COMSOL Multiphysics. The model adapts the acoustic-fluid interaction framework established by (7), (8), (9) for compressible gas mixtures. The model couples the acoustic pressure field with acoustic radiation force, gas advection, and diffusion. The gas mixtures were modeled as ideal gases using the Thermodynamics module by COMSOL. The COMSOL database was used to determine the thermodynamic properties of the different gases.

The effect of acoustic field on different gases. Movie S8 shows FEM simulations of gas dynamics in air under the acoustic field. To compare the dynamics of gases with different physical properties under identical acoustic conditions, gas columns with the diameter of 5 mm, corresponding to the diameter of the experimental tube, were initialized with the same lateral offset equal to $\lambda/5$ from the center of the acoustic field and subjected to the same m = 0 EBAF configuration shown in the bottom-left panel in the "Field on" box. The simulations revealed different stability behavior for gases with different acoustic properties. The investigated denser than air gases (air at 1°C, N_2O, CO_2, and Xe) exhibited shifting towards EBAF axis and retaining by the field. In contrast, the investigated lighter than air gases (H_2, He, CH_4, and air at 200°C) did not exhibit stable confinement and progressively moved away from the central region. These results are consistent with the experimental observations and indicate that the guidance behavior depends on the acoustic properties of the gas.

Diffusion of the gases. The bottom-left panel of Movie S8 also shows the temporal evolution of the N_2O gas density in the absence of the acoustic field. The dashed box shows the corresponding full simulation for N_2O with the acoustic field activated. In the absence of the acoustic field, the N_2O stream spreads due to molecular diffusion, with its width increasing from approximately 5.2 mm to 9.9 mm in 0.4 s, measured at a level corresponding to 33% of the difference between the maximum and minimum density at each time point. When the $m = 0$ EBAF is activated, the corresponding width increases to approximately 9.2 mm over the same time interval. The close agreement between these values suggests that the acoustic field does not substantially modify the natural molecular diffusion.

Acoustic radiation pressure on air-gas interface. In movie S8 also the graph is shown of the calculated acoustic radiation pressure at a sharp interface between air and a gas as a function of the gas density and sound speed normalized to their values in room-temperature air. The radiation pressure was calculated using the expression given, see for example (10) section 67. The direction of the force on a gas-gas interface depends on the incidence direction, the graph showing the case of the wave traveling from air to a gas. The incident pressure amplitude was set to $10^{-2}\rho_{\mathrm{air}}c_{\mathrm{air}}^2$. Positive radiation pressure corresponds to a force directed from air toward the gas, whereas negative radiation pressure corresponds to a force directed from the gas toward air. The white boundary corresponds to zero radiation pressure and therefore separates the parameter regions in which the radiation force acts in opposite directions. The dashed line indicates equal acoustic impedance, $Z = Z_{air}$. For the investigated gases, the direction of the radiation force was independent of which gas was considered as the incident medium. The parameter space where the sign of the force depends on the incidence direction is denoted with striped region. The force direction can be separated in the parameter space using density, sound speed, or acoustic impedance. The locations of the investigated gases are indicated in the parameter space, showing that the gases exhibiting guidance are located in the region where the radiation force acts outward the central acoustic field, whereas the investigated lighter than air gases are located in the region where the force acts in the opposite direction.

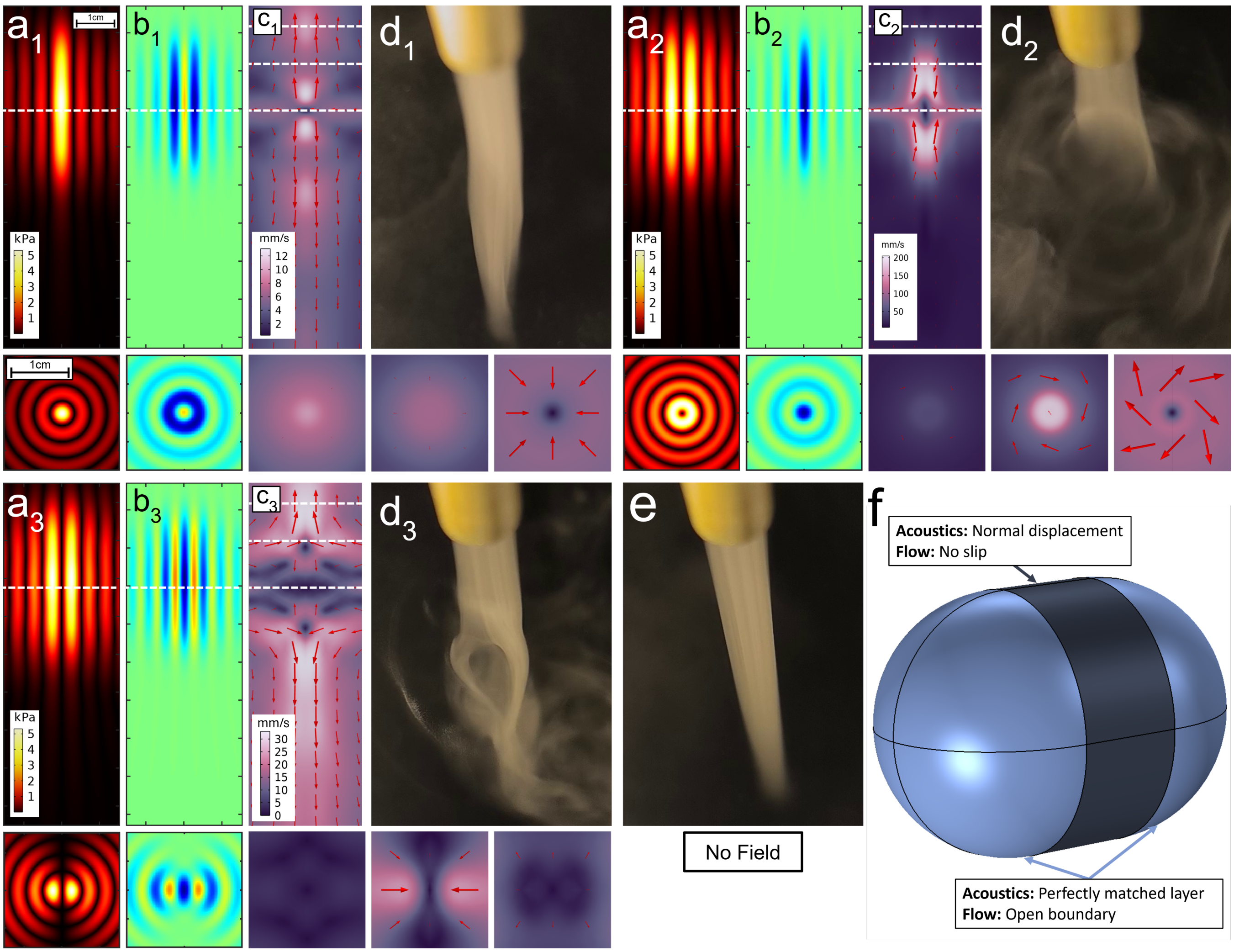

Fig. S7. (a1-a3) EBAFs of modes $m = 0, 1, \pm 1$, respectively, generated by the cylindrical section of the PAT. (b1-b3) Corresponding Gor'kov potentials. (c1-c3) Acoustic streaming distributions of these fields. Cross-sections of the streaming field are shown at planes corresponding to the dotted lines: the left cross-section corresponds to the entrance of the PAT, the middle to one-quarter of the cylindrical section length, and the right to its midpoint. (d1-d3) Experimental images showing the behavior of water aerosols in the corresponding fields. (e) Water aerosol stream without the acoustic field. (f) FEM simulation geometry.

FEM particle tracing

In the experiments, particle behavior exhibited a strong dependence on particle size. To gain further insight into this process, particle dynamics were investigated numerically using a two-dimensional FEM particle tracing simulation. In the particle tracing model, the motion of particles was computed under the action of the following forces:

ARF calculation. The acoustic radiation force acting on a particle was modeled under the assumption of an ideal fluid and a particle radius much smaller than the acoustic wavelength, $r_p \ll \lambda$. Using the Gor'kov approximation (11), the force can be expressed as:

$$\mathbf{F}^{\mathbf{ARF}}(\mathbf{r}) = 2\pi r_p^3 \left[\frac{1}{3}\kappa_S Re\left(f_0 p(\mathbf{r})^* \nabla p(\mathbf{r})\right) - \frac{1}{2}\rho Re\left(f_1 \mathbf{v}(\mathbf{r})^* \cdot \nabla \mathbf{v}(\mathbf{r})\right)\right], \quad [10]$$

where $p(\mathbf{r})$ and $\mathbf{v}(\mathbf{r})$ are the complex amplitudes of the acoustic pressure and particle velocity, respectively, at the position specified by the coordinate vector $\mathbf{r}$, r_p is the particle radius, $\kappa_S = \frac{1}{\rho c^2}$ is the isentropic compressibility of the fluid. The dimensionless coefficients f_0 and f_1, which characterize the relative compressibility and density of the particle with respect to the surrounding fluid, are defined as:

$$f_0 = 1 - \frac{\rho c_0^2}{\rho_p c_l^2} \frac{1}{1 - \frac{4c_s^2}{3c_l^2}}, \qquad f_1 = 2\frac{\rho_p - \rho}{2\rho_p + \rho}, \quad [11]$$

where ρ_p is the density of the particles, c_l and c_s are the longitudinal and shear sound speeds in the particle material, respectively. In the approximation used, the acoustic field is assumed to remain undisturbed by the presence of the particles.

Stokes' drag calculation. Fluid drag force may be modeled by (12, 13):

$$\mathbf{F}^{\mathbf{D}}(\mathbf{r}) = -\frac{1}{2} A_p \rho_f C_{Dv}(Re_p)\left(\mathbf{u}(\mathbf{r}) - \mathbf{u}_p(\mathbf{r})\right) \quad [12]$$

where A_p is the particle cross-sectional area, ρ_f is the air density, $\mathbf{u}(\mathbf{r})$ is the local fluid velocity, and $\mathbf{u}_p$ is the particle velocity. The function C_{Dv} is the drag coefficient times $|\mathbf{u}(\mathbf{r}) - \mathbf{u}_p(\mathbf{r})|$, given by:

$$C_{Dv} = \frac{24}{k_{Re}}(1 + 0.15 Re_p^{0.687}); \quad [13]$$

where $k_{Re} = 2\rho_f r_p/\mu_f$ is the constant relating the particle Reynolds number and its relative speed with the air flow $Re_p = k_{Re}|\mathbf{u}(\mathbf{r}) - \mathbf{u}_p(\mathbf{r})|$. Eq. 13 produces accurate results when Re_p is between 1 and 1000 (13). The value μ_f is the air viscosity. The drag coefficient equation is calculated this way to prevent a division by zero when $|\mathbf{u}(\mathbf{r}) - \mathbf{u}_p(\mathbf{r})|$ is zero.

Not considering the second term in Eq. 13 and simplifying gives Stokes' drag law:

$$\mathbf{F}^{\mathbf{D}}(\mathbf{r}) = -6\pi\mu_f r_p\left(\mathbf{u}(\mathbf{r}) - \mathbf{u}_p(\mathbf{r})\right), \quad [14]$$

In the present approximation, the streaming flow field is assumed to be unaffected by the presence of particles and is determined solely by the acoustic pressure distribution $p(\mathbf{r})$.

For the Stokes' drag calculation, acoustic streaming was simulated in 2D. The approach followed the methodology of the 3D streaming simulations, with the acoustic field obtained by solving the Helmholtz equation in two dimensions. The circular boundary was excited using a normal displacement condition, and the phase distribution on the radiating boundary was prescribed using the EBAFs generation algorithm. The amplitude was scaled such that the maximum pressure matched the experimental value of 5.3 kPa. Acoustic streaming was then modeled as laminar flow with a no-slip boundary condition on the circular boundary. Viscous dissipation was included as the loss mechanism, and the coupling between the acoustic field and fluid flow was introduced through COMSOL's acoustic streaming module.

Saffman lift force calculation. In addition to the acoustic radiation force and Stokes' drag, the transverse lift force due to shear in the streaming flow can be significant for small particles. This force, known as the Saffman lift force, was included in the model and is expressed as (14):

$$\mathbf{F}_L = -6.46 r_p^2 \mathbf{L}_v \sqrt{\mu\rho\frac{|\mathbf{u} - \mathbf{u}_p|}{|\mathbf{L}_v|}}, \qquad \mathbf{L}_v = (\mathbf{u} - \mathbf{u}_p) \times [\nabla \times (\mathbf{u} - \mathbf{u}_p)] \quad [15]$$

The Saffman lift force acts perpendicular to the relative motion between the particle and fluid, causing migration across streamlines in regions with significant shear.

Particle dynamics in EBAF

The simulations were conducted for zirconia/silica microspheres with diameters ranging from 25 µm to 1 mm in the 2D fields of modes $m = 0, 1, \pm 1$ (Fig.S8a). The corresponding acoustic streaming was simulated and exhibited qualitatively similar behavior to the 3D case, with the $m = 1$ field producing a strong rotational streaming component of significantly larger amplitude compared to the modes $m = 0, \pm 1$ (Fig.S8b). Although the absolute streaming magnitudes differed from the 3D results—being larger for $m = 1$ and smaller for $m = 0, \pm 1$—the relative trends remained consistent.

The particle tracing results (Fig.S8c) revealed the following:

1. Mode $m = 1$ – Particles smaller than 150–200 µm were ejected from the field center within 0.5 s, a timeframe sufficient for them to reach streaming velocities while larger particles were unaffected by streaming over the same timescale and remained aligned with the field axis. These results agree with experimental observations: zirconia/silica powder particles 100 µm were ejected by the $m = 1$ field, whereas millimeter-scale liquid droplets 1–2 mm remained guided along the axis.

2. Mode $m = 0$ – Particles smaller than 25–50 µm were trapped in the first nodal ring, while larger particles were expelled from the central focus and ejected away from the field center. This is consistent with experiments, where aerosols were guided along the tubular region surrounding the central maximum (Fig.4f) whereas larger particles were chaotically scattered around the center.

3. Modes $m = \pm 1$ – No significant differences in particle dynamics were observed across the modeled particle sizes. While this cannot explain the weak guiding observed for aerosols, it is consistent with the experimental finding that both powder particles and liquid droplets were stably guided in these fields.

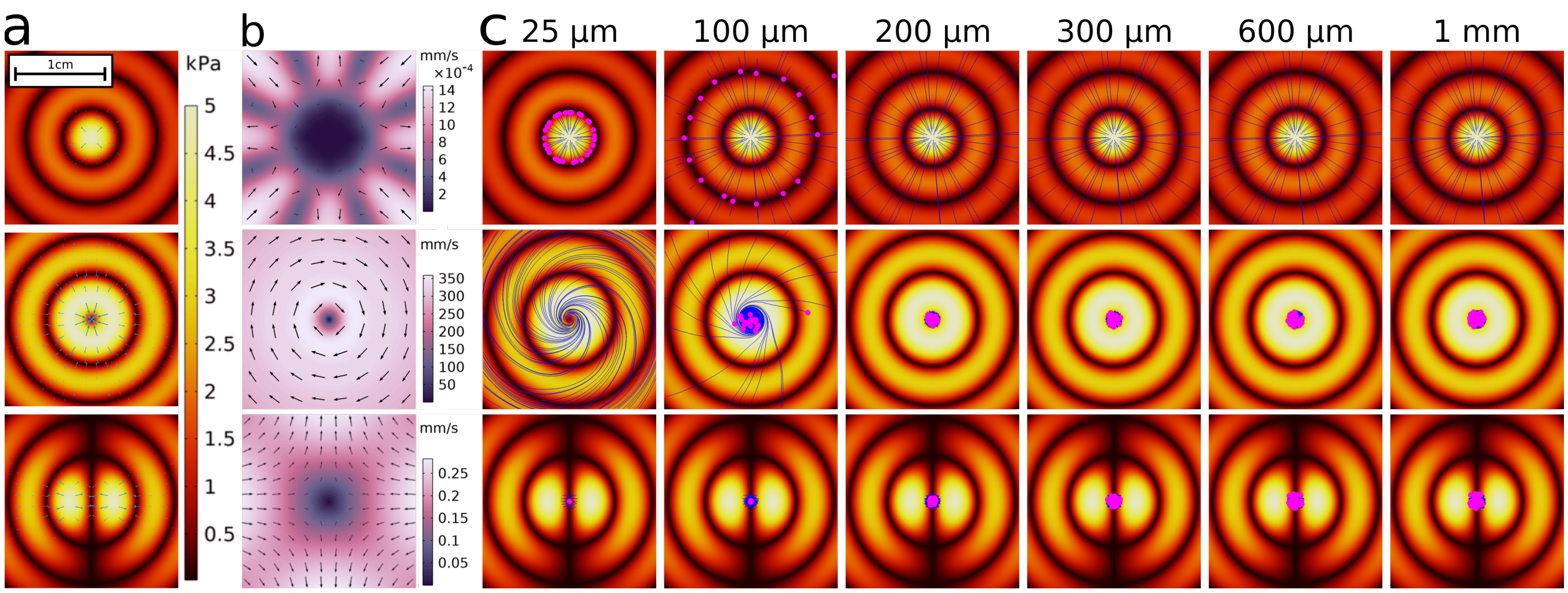

Fig. S8. Simulation of particle motion in 2D. (a) Acoustic pressure amplitude distributions for modes $m = 0, 1, \pm 1$. Arrows indicate the corresponding distributions of ARF acting on particles. (b) Corresponding acoustic streaming velocity fields are simulated for the same modes. (c) Simulated particle trajectories under the combined action of Stokes drag and acoustic radiation force (ARF) for zirconia/silica spherical particles with diameters of 25, 100, 200, 300, 600, and $1000\,\mu\mathrm{m}$.

Table S1. Summary of the effects of different EBAF modes on different types of material

Matter form	Tested material	EBAF mode	Primary/contributing mechanism	Observed behaviour	Presented results	Guidance ability
Heavier than air gases	N_2O,CO_2,Air1°C,HFO	0	ARF at the gas–air interface	Gas stream followed the EBAF trajectory	Fig. 1l, Fig. 4c, Movie S4 0:51, Movie S7	Excellent
	N_2O	1	Acoustic streaming that entrains the gas flow	Gas stream scattered	Fig. 4d	No
		±1	ARF at the gas-air interface	Stream was deflected in the correct direction but did not reach the target displacement	–	Low
Lighter than air gases	He, Air 200°C,	0	Mechanism not determined	Gas stream was not deflected and scattered randomly	Movie S6, Movie S7	No
		1	Flow entrainment	Gas stream scattered randomly	–	No
		±1	Mechanism not determined	Gas stream was not deflected and scattered randomly	–	No
Aerosols	Water mist	0	ARF on droplets + entrainment by cold-air flow steered by ARF at the cold-air–ambient-air interface	Mist followed the EBAF trajectory	Fig. 1k, Fig. 4f, Movie S4 0:21	Excellent
		1	Acoustic streaming that entrains the particles	Mist particles moved in a circular trajectory	Fig. 4g, Movie S4 0:05	No
		±1	ARF on droplets + entrainment by cold-air flow steered by ARF at the cold-air–ambient-air interface	Mist stream was deflected toward the correct direction but did not fully follow the field path	Movie S4 0:10	Low
Liquids	Continuous water stream	0	ARF on the liquid stream	Stream fragmented and was uncontrollably expelled from EBAF	–	No
		1	ARF on the liquid stream	Stream followed the EBAF path	Fig. 1h–i, Movie S3 0:00 and 0:16	Excellent
		±1	ARF on the liquid stream	Stream followed the EBAF path, but its maximum displacement was 3 mm shorter than for $m = 0$	–	Good
	Water droplets, $d \approx 2$mm	0	ARF on droplets	Droplets were expelled from the EBAF	–	No
		1	ARF on droplets	Droplets followed the EBAF path	Fig. 3d, Movie S3 0:36	Excellent
		±1	ARF on droplets	Droplets followed the EBAF path, but their maximum displacement was 4 mm lower than for $m = 0$	Fig. 3e	Good
Powders ($d < 200$ μm)	100-μm zirconia/silica microspheres; 100-μm fluorescent green polyethylene microspheres	0	ARF on particles	Powder was uncontrollably expelled from the EBAF	–	No
		1	Particle entrainment by acoustic streaming	Particles moved in a circular trajectory	Fig. 3g, Movie S3 0:56	No
		±1	ARF on particles	Powder followed the EBAF path	Fig. 1j, Fig. 3h, Movie S1 2:03, Movie S3 1:16	Excellent
Large particles ($d > 200$ μm)	0.8-mm plastic beads	0	ARF on particles	Particles were uncontrollably expelled from the EBAF	–	No
		1	ARF on particles	Particles followed the EBAF path, but a substantial fraction fell out of the EBAF	–	Good
		±1	ARF on particles	Particles followed the EBAF path, but a substantial fraction fell out of the EBAF	Movie S3 1:41	Good
Ionized air	Tesla-coil discharge	0	ARF on heated gas	Discharge followed the EBAF path	Fig. 1m, Fig. 2h–k, Fig. 4i, Movie S4 1:25 and 1:46	Excellent
		1	Mechanism not determined	Discharge path was random	Fig. 4j	No
		±1	Mechanism not determined	Discharge path was random	–	No

The table presents a summary of the observed effects of guiding different types of matter using various EBAF modes.

For solid particles and liquid streams, no pronounced qualitative dependence on material density was observed within the investigated range, whereas particle size showed a stronger influence on the guidance efficiency and on the suitability of different EBAF modes. In particular, the simulations indicate that particles larger than approximately 200μm can be more effectively guided using the $m = 1$ mode, whereas smaller particles are more effectively guided using the $m = \pm 1$ mode. For gaseous media, a stronger dependence on the acoustic properties of the gas was observed: gases denser than air were successfully guided using the $m = 0$ mode, while the investigated gases lighter than air were not guided by any EBAF modes.

Other effects, such as buoyancy and initial flow momentum, may also contribute to the observed trajectories. In the experiments conducted, buoyancy may be particularly important for gas and aerosol flows due to their relatively low density compared to ambient air. Initial flow momentum may be important for continuous liquid, gas, and aerosol flows, which were delivered with non-zero initial velocity, as opposed to powders, beads, and droplets, which were delivered with near-zero initial velocity. These effects may manifest as deviations from the target trajectory or as material escape from the EBAF during propagation. However, these factors were not independently varied and were not quantified in this study.

Particle displacement using EBAF

The experiment was conducted with zirconia/silica particles of 100 µm diameter. The particles were guided by EBAFs with a parabolic trajectory from an extraction nozzle to a sheet of grid paper placed 1 cm below the PAT. The field was fixed at the nozzle position and displaced to a prescribed offset at the opposite end of the PAT (Fig.3b). The acoustic fields were calculated using the EBAFs generation algorithm and reconfigured over the range of 0–15 mm in 1 mm steps with 0.5 or 1 s between changes. Particle displacement from the free-fall trajectory (no-field case) was measured using video analysis software. The experiment was repeated for three driving voltage peak-to-peak amplitudes applied to the transducers: 20, 25, and 30 V. The video of the experiment for 30 V is shown in Movie S3.

The results demonstrated a linear relationship between the particle displacement and the prescribed field displacement within the operational range. Here, the operational range is defined as the maximum distance at which particles could be reliably displaced before dropping out of the field near the PAT exit. As shown in Fig.3b, the operational range strongly depended on the pressure amplitude, which scaled linearly with the applied driving voltage pp amplitude. At 30 V, the maximum displacement reached 11 mm; at 25 V, it was 8 mm; and at 20 V, it was 5 mm. Beyond the operational range, particles left the EBAF before reaching its end and fell uncontrollably.

Video processing of powder stream location. The experimental final horizontal displacements were obtained using open source computer vision library OpenCV (cv2) (15) and numpy python libraries to analyze videos showing the paths of the zirconia/silica 100 µm particles.

For pre-processing the videos, they where changed to black and white, and the background grid in the video was rotated to be parallel with the video frame. An additional background image was obtained without powder. Then for both this background frame and all the other frames in the middle of the time intervals, a row of pixels was extracted, at the height where the powder stream hits the background grid.

For this set of 1D data, the following steps were executed:

- The background 1D data was subtracted, leaving the data due to the increase in brightness of the powder (data range is zero to one, with one being white)
- A moving average filter with a size 61 pixels was applied
- The remaining background noise was set to zero below a brightness of 0.04
- The position of the powder stream was estimated by finding the 'center of particle mass' of the data, with the pixel location being the distance and the zero to one intensity being the mass of the particle

After applying these steps to the frames for each time interval, the positions in pixels where subtracted from the zero pixel position determined by the grid in the frames and using a factor to convert this pixel distance to a distance in mm, obtained from the background grid, with a known grid size of 1 mm.

Material stream tracking. In the videos of powder (Movie S3, 1:27 min), liquid stream (Movie S3, 0:15 min), gas streams (Movie S4, 0:50 min), CO2, N2O and Helium (Movie S7) the position of the material stream was determined by calculating the signal-weighted center (centroid) along a predefined tracking line. For each video frame, the centroid position was calculated as

$$y_c = \frac{\sum_{i=1}^{N} \left(S_i - S_i^0\right) y_i}{\sum_{i=1}^{N} \left(S_i - S_i^0\right)}, \quad [16]$$

where i is the pixel index along the tracking line, N is the total number of pixels along the line, S_i is the recorded signal intensity at pixel i, S_i^0 is the corresponding background signal from a reference frame without the material stream, and y_i is the spatial coordinate of pixel i along the tracking line.

Background subtraction was applied to minimize the contribution of static features and inhomogeneities that were not associated with the material stream. The tracking line was positioned outside the active area of the phased array, in the near vicinity of the array exit. The location of the tracking line is indicated by the blue line in the corresponding video. The centroid indicated by the green dot.

In order to track Cold Air (Movie S7), due to the lack of sufficient contrast between the air and the background, multiple tracking lines were used along the direction of the stream. The centroid at the exit of the array was then estimated by fitting a third-degree polynomial with the centroid positions obtained from all the tracking lines and extrapolating the fit to the array exit. Finally, for tracking of water droplets (Movie S3 0:49), because the drop is not a continuous stream, for certain frames, no water drop crosses the tracking line, so a manual tracking was performed using the tracking tool call Tracker.

Quantitative assessment of material guidance. To quantitatively assess the guidance performance, image based tracking was applied to video recordings of the powder, liquid, droplet, and gas stream experiments. Because the experiments were recorded using a single-perspective optical setup, full three-dimensional reconstruction of the material trajectories along the entire guidance path was not feasible. Therefore, quantitative tracking was performed at the array-exit plane, where the streams intersected a reference scale grid. The experimentally measured position of each material stream was compared with the target position prescribed by the corresponding EBAF displacement.

1. Trajectory accuracy. The maximum positional deviations from the intended EBAF displacement at the array exit plane were approximately 2 mm for the powder (Movie S3, 1:19), 3 mm for the liquid stream (Movie S3, 0:15), 8 mm for the droplets (Movie S3, 0:36), and 2 mm for the gas stream (Movie S4, 0:53).

2. Operational steering bounds. Complete material retention along the acoustic trajectory was maintained up to lateral displacements of 11 mm for powder, 16 mm for the liquid stream, 15 mm for droplets, and 9.5 mm for the gas stream. At larger displacements, rapid loss of material from the guided trajectory was observed. For the droplet experiment, a relatively large deviation between the measured and target positions was observed for target displacements between 5 and 15 mm. This deviation is attributed to imperfect alignment between the droplet ejection needle and the phased array, which caused the droplets to fall out from the EBAF at specific lateral displacements.

3. Material retention. Within the established operating limits, liquid streams remained confined along the prescribed EBAF trajectories. Analysis of the illuminated fluorescent water (Movie S1, 2:04 left panel) showed that only individual droplets were lost from the guided trajectory during changes in the EBAF direction, while the most of droplets remained successfully guided to the target location. Powder streams showed only minor visual fallout of individual grains under static EBAF (as seen in Movie S1 2:04). However, when the EBAF changed its trajectory a portion of the powder was lost. The gas stream remained visually confined along the full guidance path, although video analysis showed that its width increased from approximately 5 mm at the tube outlet to approximately 10 mm at the array exit.

Throughout the steering experiments, droplets exhibited lower guidance accuracy than the other tested materials, with larger deviations from the target trajectory. This may be related to the relatively large droplet size compared with the width of the EBAF, making the droplets more sensitive to the initial ejection conditions, including their initial position and velocity.

Simulation of particles guiding in EBAF. The particles were tracked through the acoustic field by applying to them the Gor'kov, drag forces and gravity after each time step using the forward Euler method. These forces were computed for each particle at each spatial location. The Gor'kov force at a point was calculated using Eq.10. The only approximation error in this case comes from the Euler method integration of the particle motion, since Eq.10 uses the transducer model derivatives, which can be computed exactly using automatic differentiation up to machine precision. The drag force was calculated using Eq.12. After running the simulations, the maximum speed seen by the particles was about 1 m/s. Using this value, the maximum Re_p value was calculated to be less than 7, which means either Eq.13, or the Stokes' approximation for low Re_p, using only the first term of Eq.13, could have been used.

The simulation was performed for EBAFs with a parabolic trajectory, generated using the conical section of the PAT (Fig.3a) and drive signals computed via the EBAFs generation algorithm. The parabolic field shift at the end of the PAT was varied within the range of 0–12 mm. Each numerical test included 200 particles, normally distributed spatially, with a standard deviation of 0.4 mm, a normally distributed sideways speed with a standard deviation of 15 mm/s, and an initial downward speed of 20 mm/s. The average displacement of particles from the field axis was evaluated at a position 10 mm below the end of the PAT. The numerical predictions agreed with experimental measurements of particle displacement at the PAT exit (Fig.3b), with deviations below 20% in the operational range where particles followed the channel.

Scalability and practical implementation of the system

The scalability of the proposed system can be considered in terms of both guidance distance and material processing throughput. Longer guidance distances can be achieved by increasing the active length of the phased array through the addition of further array segments. This modular approach allows the operational region to be extended without fundamentally changing the operating principle of the system. The achievable throughput can potentially be increased through optimization of the acoustic pressure amplitude, material extraction conditions, and EBAF trajectory for a particular application.

The transducers used in the present system are commercially available and relatively inexpensive, with a unit cost of approximately $0.45 USD. The array operates at approximately 40 kHz, allowing the use of relatively simple driving electronics without the high-frequency signal-generation and protection requirements associated with MHz-range ultrasonic systems. The control electronics are based on commercially available FPGAs. The relatively long acoustic wavelength also allows for lower mechanical assembly tolerances. In the present system, an installation accuracy of approximately 0.1 mm was sufficient. The array housing can be manufactured using conventional 3D-printers.

The complete experimental system, including the transducers, driving electronics, housing, and operating components, cost less than approximately $500 USD for the prototype, corresponding to an average system cost of approximately $2 USD per active channel. The average electrical power consumption of the complete system during the experiments was less than 100 W. Further optimization of the electronics and mechanical assembly, together with modular production, could reduce the cost per channel for larger-scale implementations.

Overall, the combination of modular architecture, commercially available components, relatively low power consumption, and low prototype cost make the proposed approach potentially attractive for applications requiring programmable non-

contact material delivery and processing, including additive manufacturing, bioprinting, non-contact material distribution, and non-contact welding.

Applications of discharge guidance

In the present implementation, a Tesla-coil discharge was used as a convenient source of ionized gas, and approximately 15 s was required to melt and join two plastic pieces with a width of 12 mm and a thickness of 1 mm (Movie S5). The discharge remained confined to the prescribed EBAF trajectory with deviations of approximately 2 mm. Unlike conventional plasma arc welding, which employs a high-current plasma discharge, the present Tesla-coil discharge is low power and is therefore not suitable for efficient metal welding. However, the demonstrated ability to acoustically position the ionized discharge provides a potential route toward non-contact steering of higher-power plasma arcs. Integration with high-current plasma sources could potentially enable electronically programmable positioning of the heat source without mechanical movement of the welding torch.

Movie S1. This video summarizes the study, illustrating the principle of Elongated Bessel-like Acoustic Fields (EBAFs) generation using PATs, and the resultant continuous guidance of material in various states along three-dimensional paths.

Movie S2. 3D field distributions comparing experimental scans with numerical simulations of EBAFs for m=1 straight (0:00-0:31), m=1 tilted (0:31-1:02), m=2 straight (1:02-1:33), m=2 tilted (1:33-2:05). The trajectory curving pf EBAFs of the modes $m = \pm 1$, $m = 1$, and $m = 0$ is shown in 2:05-2:16.

Movie S3. Demonstration of the guidance of laminar liquid stream (0:00-0:34), droplets (0:34-0:53), powder (0:53-1:40), and solid particles (1:40-1:47) via different EBAF modes and identifies the optimal mode for each case.

Movie S4. Guidance of water aerosol (0:00-0:46), N_2O (0:46-1:20), and ionized air generated using Tesla coil discharge (1:20-2:03), demonstrating that the optimal EBAF mode identified for aerosols provides effective guidance of all these gaseous media.

Movie S5. Demonstration of application scenarios enabled by acoustic guidance, including controlled material deposition (0:00-0:10), non-contact welding processes (0:10-0:24), gas capturing (0:24-0:35), directed liquid and powder transport (0:34-0:49), and liquids mixing (0:49-1:02).

Movie S6. 0:00 - 0:12 Comparison of the effect of the acoustic field on room-temperature air (20°C) and the heavy gas HFO-1234ze under identical acoustic conditions. 0:12 - 0:22 Comparison of the effect of the acoustic field on heated (200°C) air and the heavy gas HFO-1234ze under identical acoustic conditions.

Movie S7. Steering of CO_2, N_2O, cold air at 1°C, and He under identical $m = 0$ EBAF conditions, with the acoustic trajectory displaced by 10 mm in both upward and downward directions.

Movie S8. FEM simulations of the dynamics of gases with different acoustic properties under identical $m = 0$ EBAF conditions. The upper panels show the temporal evolution of gas density and flow velocity for gases lighter than air (H_2, He, CH_4, and air at 200°C) and gases denser than air (air at 1°C, N_2O, CO_2, and Xe). The simulations demonstrate stable confinement and restoring motion toward the EBAF axis for the investigated denser than air gases, whereas the lighter than air gases progressively move away from the central region. For comparison, the movie also shows the evolution of He in the absence of the acoustic field, where only diffusion is considered, together with the corresponding simulation with the acoustic field. The movie also shows the calculated normalized acoustic radiation pressure at a sharp gas-air interface as a function of gas density and sound speed. The map illustrates the dependence of the ARF direction on the combined acoustic properties of the gas and surrounding air. The locations of the investigated gases are indicated, showing correspondence between the radiation-pressure regions and the experimentally observed guidance and no-guidance behavior. For further details of the radiation-pressure map and its interpretation, see the section "FEM simulation of gas dynamics."

References

1. EG Williams, *Fourier acoustics: sound radiation and nearfield acoustical holography.* (Academic Press, NY), (1999).
2. OA Sapozhnikov, SA Tsysar, VA Khokhlova, W Kreider, Acoustic holography as a metrological tool for characterizing medical ultrasound sources and fields. *The J. Acoust. Soc. Am.* **138**, 1515–1532 (2015).

3. G Kino, *Acoustic Waves: Devices, Imaging, and Analog Signal Processing*, Prentice-Hall Contemporary Topics in Accounting Series. (Prentice-Hall, Upper Saddle River, NJ), (1987).
4. OT Von Ramm, SW Smith, Beam steering with linear arrays. *IEEE Transactions on Sonics Ultrason.* **30**, 89–101 (1983).
5. P Martínez, J Ruiz, Íñigo Martín, M Lucas, Experimental study of an ultrasonic mist generator as an evaporative cooler. *Appl. Therm. Eng.* **181**, 116057 (2020).
6. R Tuckermann, B Neidhart, EG Lierke, S Bauerecker, Trapping of heavy gases in stationary ultrasonic fields. *Chem. physics letters* **363**, 349–354 (2002).
7. JT Karlsen, P Augustsson, H Bruus, Acoustic force density acting on inhomogeneous fluids in acoustic fields. *Phys. Rev. Lett.* **117**, 114504 (2016).
8. JH Joergensen, H Bruus, Theory of pressure acoustics with thermoviscous boundary layers and streaming in elastic cavities. *The J. Acoust. Soc. Am.* **149**, 3599–3610 (2021).
9. VK Rajendran, S Jayakumar, M Azharudeen, K Subramani, Theory of nonlinear acoustic forces acting on inhomogeneous fluids. *J. Fluid Mech.* **940**, A32 (2022).
10. L LANDAU, E LIFSHITZ, Chapter viii - sound in *Fluid Mechanics (Second Edition).* (Pergamon), Second edition edition, pp. 251–312 (1987).
11. LP Gorkov, On the forces acting on a small particle in an acoustical field in an ideal fluid. *Sov. Physcis Doklady* **140** (1962).
12. K Hiltunen, et al., Multiphase flow dynamics. *Theory Numer. Tech. Rep* **722** (2009).
13. G Ahmadi, Hydrodynamic forces - ME 537: Fluid Mechanics of Aerosols course notes at Clemson University (https://sites.clarkson.edu/gahmadi/wp-content/uploads/sites/28/2020/06/ME537-1_2Drag.pdf) (2026).
14. PG Saffman, The lift on a small sphere in a slow shear flow. *J. Fluid Mech.* **22** (1965).
15. G Bradski, The OpenCV Library. *Dr. Dobb's J. Softw. Tools* (2000).
